# Intravenous BCG-mediated protection against tuberculosis requires CD4^+^ T cells and CD8α^+^ lymphocytes

**DOI:** 10.1084/jem.20241571

**Published:** 2025-02-06

**Authors:** Andrew W. Simonson, Joseph J. Zeppa, Allison N. Bucsan, Michael C. Chao, Supriya Pokkali, Forrest Hopkins, Michael R. Chase, Andrew J. Vickers, Matthew S. Sutton, Caylin G. Winchell, Amy J. Myers, Cassaundra L. Ameel, Ryan J. Kelly, Ben Krouse, Luke E. Hood, Jiaxiang Li, Chelsea C. Lehman, Megha Kamath, Jaime Tomko, Mark A. Rodgers, Rachel Donlan, Harris Chishti, H. Jacob Borish, Edwin Klein, Charles A. Scanga, Sarah M. Fortune, Philana Ling Lin, Pauline Maiello, Mario Roederer, Patricia A. Darrah, Robert A. Seder, JoAnne L. Flynn

**Affiliations:** 1 Department of Microbiology and Molecular Genetics, University of Pittsburgh School of Medicine, Pittsburgh, PA, USA; 2 Center for Vaccine Research, University of Pittsburgh School of Medicine, Pittsburgh, PA, USA; 3 Vaccine Research Center, National Institute of Allergy and Infectious Diseases (NIAID), National Institutes of Health (NIH), Bethesda, MD, USA; 4 Ragon Institute of MGH, MIT, and Harvard, Cambridge, MA, USA; 5 Department of Immunology and Infectious Diseases, Harvard T.H. Chan School of Public Health, Boston, MA, USA; 6 Division of Animal Laboratory Resources, University of Pittsburgh School of Medicine, Pittsburgh, PA, USA; 7 Department of Pediatrics, Children’s Hospital of the University of Pittsburgh of UPMC, Pittsburgh, PA, USA; 8 Broad Institute of MIT and Harvard, Cambridge, MA, USA

## Abstract

Tuberculosis (TB) is a major health burden worldwide despite widespread intradermal (ID) BCG vaccination in newborns. We previously demonstrated that changing the BCG route and dose from 5 × 10^5^ CFUs ID to 5 × 10^7^ CFUs i.v. resulted in prevention of *Mycobacterium tuberculosis* (Mtb) infection and TB disease in highly susceptible nonhuman primates. Identifying immune mechanisms protection following i.v. BCG will facilitate development of more effective vaccines against TB. Here, we depleted lymphocyte subsets prior to and during Mtb challenge in i.v. BCG–vaccinated macaques to identify those necessary for protection. Depletion of adaptive CD4 T cells, but not adaptive CD8αβ T cells, resulted in loss of protection with increased Mtb burdens and dissemination, indicating that CD4 T cells are critical to i.v. BCG–mediated protection. Depletion of unconventional CD8α-expressing lymphocytes (NK cells, innate T cells, and CD4^+^CD8α^+^ double-positive T cells) abrogated protection in most i.v. BCG–immunized macaques, supporting further investigation into which of these cell subsets contribute to protection after vaccination.

## Teaser

Antibody depletion of select lymphocytes in rhesus macaques demonstrated key roles for CD4^+^ T cells and CD8α^+^ lymphocytes in conferring sterilizing immunity against tuberculosis following i.v. BCG vaccination. This study established mechanisms of protection that can inform future vaccine strategies.

## Introduction

*Mycobacterium tuberculosis* (Mtb) caused over 10.8 million cases of active tuberculosis (TB) in 2023 and 1.25 million deaths, with most of the global burden of disease seen in low- and middle-income countries (World Health Organization, 2024). Efforts at reducing TB were limited by disruptions to medical infrastructure and research interests due to the COVID-19 pandemic (Falzon et al., 2023; Pai et al., 2022). To combat the ongoing TB epidemic, new vaccine approaches are critical (Knight et al., 2014; Migliori et al., 2023; Voss et al., 2018). However, despite decades of research, there are still major gaps in our understanding of the mechanisms of effective control of Mtb infection and which immune factors should be targeted in a vaccine strategy (Darrah et al., 2019; Flynn and Chan, 2022; Nunes-Alves et al., 2014), in part due to the lack of a model of full immunological control. Currently, the live attenuated Bacille Calmette–Guérin (BCG) vaccine is the only licensed vaccine in use and is administered intradermally (ID) at birth in much of the world, including high-burden regions (Zwerling et al., 2011). ID BCG confers protection against disseminated disease in children but has variable efficacy against pulmonary disease, and protection generally wanes through adolescence (Roy et al., 2014). BCG vaccination in adults has little efficacy (Martinez et al., 2022). Despite limited durable immunity against TB disease, BCG has been used for over 100 years to protect children. However, there has been a lack of success by alternative vaccine candidates and regimens to improve protection in adults (Andersen and Doherty, 2005; Soleimanpour et al., 2022).

Identifying vaccine strategies that provide protection in an animal model that recapitulates key aspects of human TB is essential to dissecting the immune factors required for prevention of infection or disease. This knowledge facilitates development of vaccines suitable for clinical trials. Nonhuman primates (NHPs) are the closest genetically related animal model of infectious disease to humans, possess similar respiratory system anatomy and closely mirror human immune system composition and function. When infected with a low dose of Mtb, NHPs develop TB with similar outcomes, spanning subclinical and active progressive disease, and pathologies, such as fully circumscribed granulomas and more extensive pathologies, to humans (Capuano et al., 2003; Lin et al., 2009). Rhesus macaques (*Macaca mulatta*), specifically, are extremely susceptible to progressive disease following Mtb infection. In the context of intervention studies, rhesus macaques therefore serve as a particularly rigorous model for evaluating vaccine efficacy, as control of an infection can be largely attributed to the immunization (Maiello et al., 2018).

Recent studies in rhesus macaques from our group have demonstrated that the efficacy of BCG was greatly enhanced by changing the administration method from the conventional low-dose ID route (Darrah et al., 2020). High-dose i.v. BCG elicited a significantly higher number of CD4 and CD8 T cells in the airways and lung tissue in macaques compared with the same dose given by ID or aerosol immunization. Following Mtb challenge, i.v. BCG vaccination resulted in 90% of animals being protected (<100 total Mtb CFUs recovered), with 60% developing sterilizing immunity. BCG i.v. is unique in providing sterilizing immunity at a high frequency in the highly susceptible rhesus macaque model of TB.

Follow-up studies investigated potential correlates of protection, which are measurable immune responses that can be used clinically to predict protective efficacy. Correlates analyses of macaques immunized with a wide dose range of i.v. BCG revealed a highly integrated and coordinated immune response in the airway, some features of which could be predicted by early (day 2) innate signatures in whole blood (Darrah et al., 2023; Liu et al., 2023). Antigen-specific cytokine-producing CD4 T cells (IL-2, TNF, IFN-γ, or IL-17) and natural killer (NK) cell numbers in the airway were among features most strongly correlated with protection. A parallel systems serology analysis of the study showed that humoral signatures, including complement-fixing IgM and NK cell–activating antibody, were associated with protection (Irvine et al., 2021, 2023, *Preprint*). Together, these data suggest that i.v. BCG elicits a multifaceted immune response that mediates high-level protection against infection and disease.

However, predictive immune correlates do not always align with the underlying mechanisms mediating protection against infection and disease. In a recent study, our group depleted B cells during and following i.v. BCG vaccination using the CD20-targeted antibody, rituximab (Wang et al., 2024). Antibody responses in the airway (bronchoalveolar lavage [BAL]) and blood (serum) are substantial following i.v. BCG vaccination (Darrah et al., 2020; Irvine et al., 2023, *Preprint*). Only a modest effect on i.v. BCG–induced protection was observed in the B cell depletion group, driven by anti-lipoarabinomannan antibody titers, and based on the data, a much larger sample size of animals would be required to see a significant reduction in protection against Mtb (Wang et al., 2024). Thus, while humoral responses that are measurable in clinical settings may serve as surrogate markers for effective responses, their requirement for protection mediated by BCG i.v. may be limited (Darrah et al., 2023; Wang et al., 2024). Furthermore, mechanisms of natural- versus vaccine-elicited protection may differ. We have shown CD4 T cells and CD8α^+^ lymphocytes play a role in limiting Mtb infection in unvaccinated cynomolgus macaques (Lin et al., 2012; Winchell et al., 2023). However, the mechanism by which i.v. BCG exerts an unprecedented level of vaccine-mediated protection in rhesus macaques has not been directly tested. Here, we used in vivo antibody-mediated depletion in rhesus macaques to define specific lymphocyte subsets contributing to i.v. BCG–induced immunity.

In this study, lymphocyte depletion began 5 mo after i.v. BCG immunization (1 mo before Mtb challenge) and continued throughout the challenge phase. Vaccinated NHPs were depleted of lymphocyte subsets using anti-CD4, anti-CD8α, or anti-CD8β rhesusized antibodies. While the CD4 receptor is expressed as a single protein on T cells, CD8 is expressed as a dimer on the surface of a wide variety of innate and adaptive immune lymphocytes. Conventional adaptive CD8 T cells generally express CD8α and CD8β as a heterodimer. In contrast, innate-like cells, such as NKs, γδ T cells, NK T cells (NKTs), and mucosal-associated invariant T cells (MAITs), are heterogeneous with respect to their expression of CD4, CD8 (αα or αβ), both (double positive, DP), or neither (double negative, DN). Thus, anti-CD8α broadly depletes most CD8^+^ innate and adaptive lymphocytes, as well as a subset of CD4 T cells that express CD8α, while CD8β depletion more selectively targets CD8β-expressing lymphocytes, the majority of which being conventional adaptive CD8 T cells. While it is not feasible to deplete each individual CD8^+^ cell subtype in NHPs, inclusion of both CD8α and CD8β depletion groups enables a general parsing of involved cellular mechanisms of protection. A companion study from our group demonstrated robust depletion of the expected subsets in the blood, airways, and tissues (including the lung) using these same in vivo antibodies in i.v. BCG–vaccinated (but unchallenged) macaques (Sutton et al., 2024). Here, we substantiate these findings in blood and tissue samples from vaccinated animals that were subsequently Mtb challenged and followed for protection. Our results demonstrate that depletion of CD4 T cells or all CD8α^+^ lymphocytes substantially diminished i.v. BCG–induced protection against Mtb. By pairing Mtb outcome data with immunological assays and Mtb bacterial barcoding, we identified spatial immune bottlenecks to controlling Mtb. Our study supports the importance of CD4 T cells in i.v. BCG–induced protection against Mtb and raises an intriguing possibility that innate CD8α^+^ lymphocytes may also play a critical role.

## Results

### Study design and lymphocytes targeted by in vivo depletion

**Figure 1.**
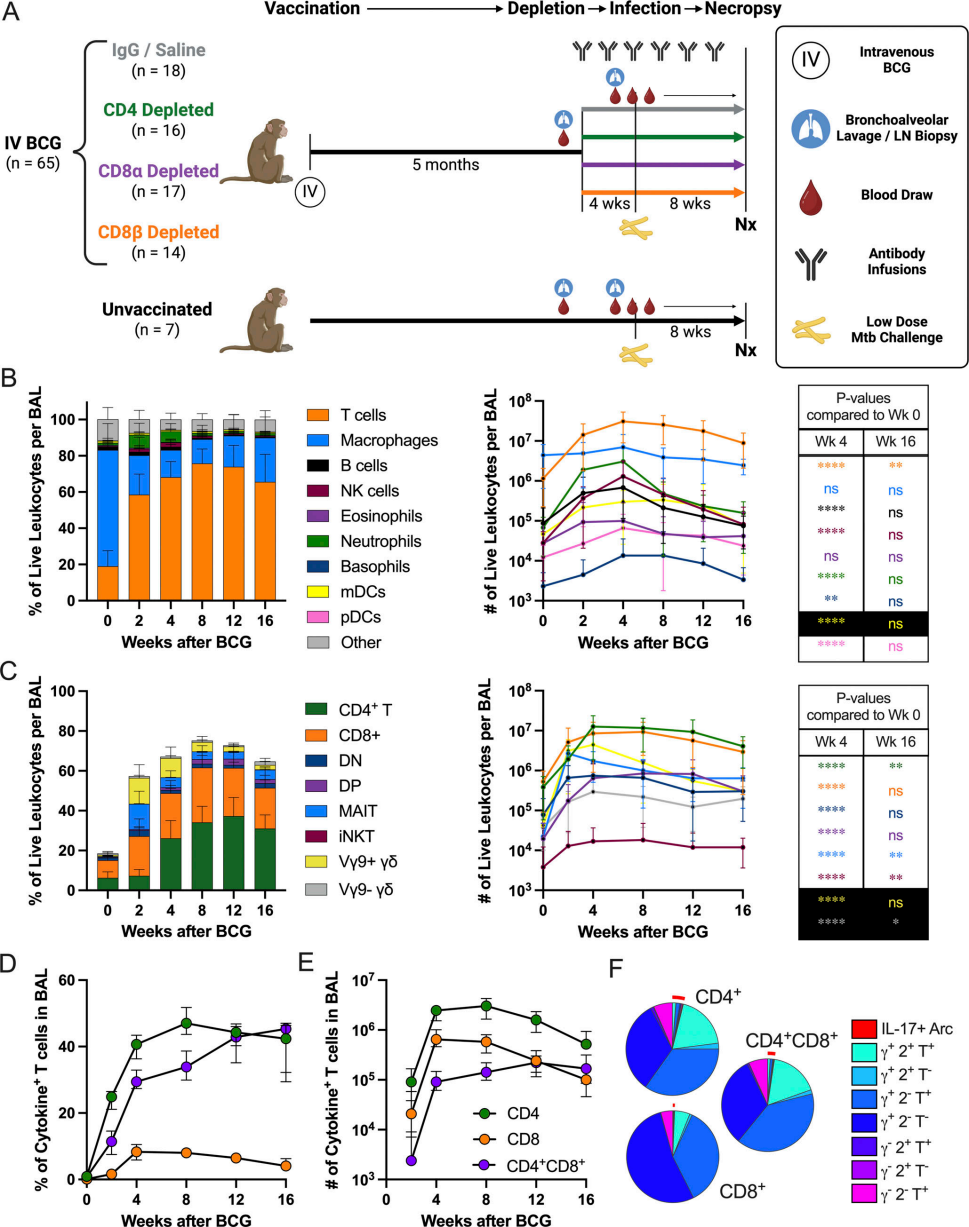
**A robust immune response is induced by i.v. BCG vaccination. (A)** Schematic outlining general timeline of the study. Made with BioRender. **(B)** Relative abundance of cell types in BAL of i.v. BCG–vaccinated macaques (*n* = 15–65) shown as the mean proportion of live leukocytes with standard deviation (left), the number of cells as mean with standard deviation (center), and table showing significant differences in number of cells in BAL using Tukey’s multiple comparison test to week 0 (right). Cell types shown are T cells (orange), macrophages (aqua), B cells (black), NK cells (maroon), eosinophils (purple), neutrophils (green), basophils (teal), mDCs (yellow), pDCs (pink), and other cells that were unclassified by flow cytometry (gray). **(C)** Relative abundance of T cell subsets in BAL of i.v. BCG–vaccinated macaques (*n* = 15–65), reported as the mean frequency of live leukocytes with standard deviation (left), number of cells as mean with standard deviation (center), and table showing significant differences in number of cells in BAL using Tukey’s multiple comparison test to week 0 (right). T cell types shown are CD4^+^ T cells (green), CD8^+^ T cells (orange), CD4^−^CD8^−^ DN T cells (teal), CD4^+^CD8^+^ DP T cells (purple), MR1 5-OP-RU^+^ MAITs (aqua), TCRVα24^+^ iNKTs (maroon), Vγ9^+^ γδ T cells (yellow), and Vγ9^−^ γδ T cells (gray). **(D and E)** Frequency (D) and number (E) of antigen-specific T cells in the BAL of i.v. BCG–vaccinated macaques (*n* = 15–65) producing IFN-γ, TNFα, IL-2, or IL-17 in response to Mtb WCL. Data in D and E are shown as background-subtracted medians with 95% confidence intervals. **(E)** Week 0 after BCG shown below the axis. **(F)** Week 4 stratification (*n* = 34) of background subtracted IFN-γ (γ), TNF (T), IL-2 (2), and IL-17 cytokine responses from BAL CD4^+^, CD8^+^, and CD4^+^CD8^+^ DP T cells with a red arc denoting IL-17^+^ T cells. ns = not significant, P ≥ 0.05; * = 0.01 ≤ P < 0.05; ** = 0.001 ≤ P < 0.01; and **** = P < 0.0001.

**Figure S1.**
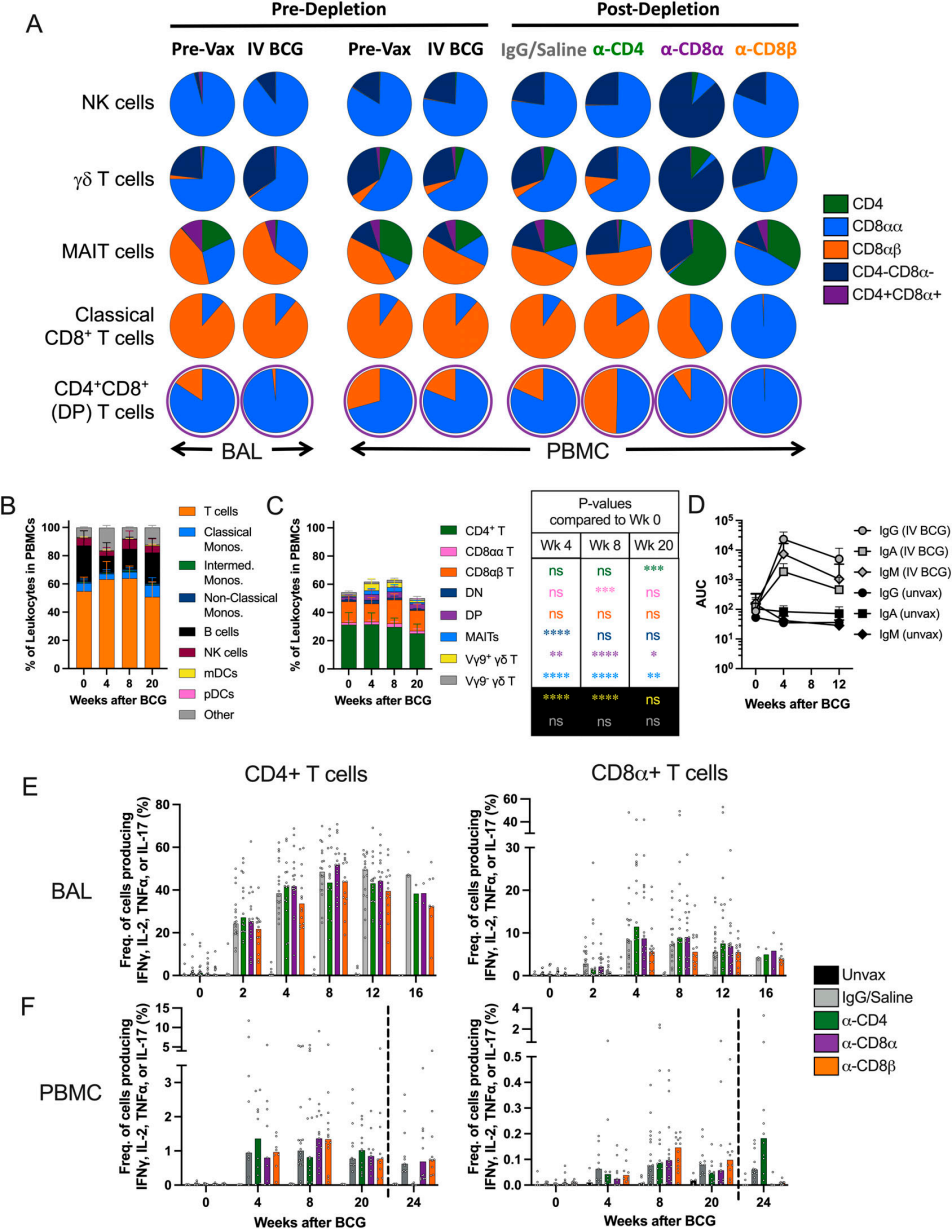
**Changes in the composition of lymphocyte cell types in blood of i.v. BCG–vaccinated macaques following cell type depletion. (A)** Frequency of common lymphocyte cell types’ CD4 and CD8α/β distributions measured by flow cytometry in BAL and PBMCs. CD4^+^ (green), CD8αα (light blue), CD8αβ (orange), CD4^−^CD8α^−^ (dark blue), and CD4^+^CD8α^+^ (purple) phenotypes are shown for each cell type. NK cells (top row), γδ T cells (second row), MAIT cells (third row), classical CD8^+^ T cells (fourth row), and CD4^+^CD8α^+^ T cells (bottom row) are shown. Columns show baseline (one BAL and three PBMCs), following vaccination prior to depletion at approximately week 13 (two BAL and four PBMCs), and the effects of each antibody-mediated depletion in PBMCs at week 20 after vaccination and week 4 after depletion (5–8). Pie charts reflect *n* = 4 BAL samples before and after vaccination, *n* = 72 pre-vax PBMCs, *n* = 65 i.v. BCG post-vax pre-depletion PBMCs, and post-depletion PBMCs from *n* = 18 IgG, *n* = 16 anti-CD4, *n* = 17 anti-CD8α, and *n* = 14 anti-CD8β-depleted NHPs. **(B and C)** Relative frequency of leukocyte (B) and T cell (C) subsets in PBMCs following i.v. BCG vaccination (*n* = 47–65), shown as mean with standard deviation (center-left). Statistics in table show the significant differences in percentage of T cell subsets in PBMCs using Tukey’s multiple comparison test to week 0 (center-right). **(D)** Antibody titers to mycobacterial antigens in concentrated BAL fluid shown as mean with standard deviation (*n* = 65). **(E and F)** Median frequency of cytokine (IFN-γ, TNFα, IL-2, and/or IL-17) producing CD4^+^ (left) and CD8α^+^ (right) T cells in BAL (E) and PBMCs (F) in response to WCL stimulation. Dotted line in F reflects when PBMC samples collected following depletion antibody administration. BAL samples: Unvax (*n* = 2–6), i.v. BCG (*n* = 3–18), α-CD4 (*n* = 2–16), α-CD8α (*n* = 2–17), and α-CD8β (*n* = 8–14). PBMC samples: Unvax (*n* = 4–7), i.v. BCG (*n* = 9–18), α-CD4 (*n* = 9–16), α-CD8α (*n* = 9–17), and α-CD8β (*n* = 8–14). ns = not significant, P ≥ 0.05; * = 0.01 ≤ P < 0.05; ** = 0.001 ≤ P < 0.01; *** = 0.0001 ≤ P < 0.001; and **** = P < 0.0001.

Rhesus macaques were vaccinated i.v. with 1.7–4.5 × 10^7^ CFUs (target dose: 5 × 10^7^) of BCG Danish (*n* = 65), as described previously (Darrah et al., 2020, 2023). 5 mo after vaccination, the NHPs were allocated to treatment groups receiving biweekly infusions of antibodies targeting CD4 (*n* = 16), CD8α (*n* = 17), or CD8β (*n* = 14) to deplete cells expressing these markers (Fig. 1 A) (Sutton et al., 2024). The final group (*n* = 18) of vaccinated animals received IgG or saline infusions as a nondepleting control. Cellular composition and function of peripheral blood mononuclear cells (PBMCs), airway cells from BAL, and peripheral LN biopsies were assessed at baseline, following vaccination and after depletion. Fig. S1 shows the expression of CD4, CD8αα, and CD8αβ on innate (NK cells, γδ T cells, and MAITs) lymphocytes and adaptive CD8α-expressing lymphocyte subsets in BAL and PBMCs. Previous studies from our group and others have shown that CD8α expression on NK cells and γδ T cells is variable across macaques and can vary in blood versus tissue (Diedrich et al., 2023; Sutton et al., 2024; Winchell et al., 2023). Generally, NK cells are predominantly CD8αα^+^ in blood and BAL, whereas γδ T cells are split between CD8αα^+^ and DN. Approximately half of MAIT cells express CD8αβ while the remainder are a mix of CD8αα^+^, CD4^+^, DN, and DP. A proportion (∼10%) of CD4 T cells in macaques are CD4^+^CD8α^+^ DP (expressing mostly CD8αα). In some settings, these DP cells are more activated and functional than their CD4 SP counterpart. Taken together, both classical CD8 T cells and a majority of innate lymphocytes are broadly targeted by anti-CD8α, whereas most classical adaptive CD8 T cells and few innate lymphocytes will be depleted by anti-CD8β (Fig. S1 A) (Grant et al., 2022). The efficiency of antibody depletion and composition of targeted cell types, plus their functions in various tissues, was reported using unvaccinated and i.v. BCG–vaccinated Mtb naïve animals in a recently published study from our group (Sutton et al., 2024).

6 mo after i.v. BCG and 1 mo after initiating depletion antibody infusions, macaques were challenged intrabronchially with a low dose (5–39 CFUs, median: 15) of genetically barcoded Mtb Erdman, as previously described (Capuano et al., 2003; Martin et al., 2017). Antibody infusions were continued biweekly for the duration of the study. The infection was monitored clinically and by serial positron emission tomography and computed tomography (PET CT) scans using ^18^F-fluorodeoxyglucose (FDG) as a PET probe for 8 wk. FDG activity indicates increased cellular metabolism, a sign of localized inflammation, and is a proxy for TB disease (White et al., 2017). Pre-Mtb challenge scans 6 mo after BCG i.v. vaccination showed negligible inflammation remaining in the lungs. Even after systemic delivery of high doses, BCG was found only at very low levels >4 wk after vaccination (Darrah et al., 2020). At necropsy 8 wk after challenge, the final PET CT scan was used as a map to identify individual granulomas and other pathologic lesions, which, along with lung lobes and LNs, were excised and homogenized into single-cell suspensions for microbiological and immunological analysis.

This study was performed in two cohorts, each with multiple BCG vaccination and Mtb challenge groups. Of note, the anti-CD8β antibody was only included in the second cohort. Specific cohort information, including vaccination and infection doses for each animal, is reported in Table S1. Control groups included i.v. BCG–vaccinated macaques (positive control for protection) treated with saline or nonspecific IgG and unvaccinated macaques (negative control). Although our previous i.v. BCG studies had a challenge phase duration of 12 wk, here we chose a challenge endpoint of 8 wk due to concern of more severe TB disease in the setting of T cell depletion.

### i.v. BCG induces robust immune responses

Similar to our prior studies, i.v. BCG vaccination increased viable cells in the airways (BAL), resulting in the T cell compartment increasing from <20% to >60% of total BAL leukocytes within 4 wk. In the first month, numbers of T cells, B cells, NK cells, neutrophils, basophils, and dendritic cells increased, but only T cells remained significantly elevated by 16 wk after BCG (Fig. 1 B) (Darrah et al., 2020, 2023). Compositional changes in PBMCs are subtle compared with BAL, but proportions of CD4^+^CD8α^+^ DP, MAIT, and Vγ9^+^ γδ T cells were increased between 4 and 8 wk after BCG (Fig. S1, B and C). IgG, IgA, and IgM antibody responses against whole-cell lysate (WCL) were confirmed in the BAL after i.v. BCG vaccination (Fig. S1 D). Of the T cell subsets captured by our flow cytometry panel, all were increased in number early after i.v. BCG, with classical CD4 and CD8 T cells comprising the largest proportions (Fig. 1 C, 4 wk). By 16 wk, CD4, MAIT, invariant NKTs (iNKTs), and Vγ9^−^ γδ T cells remained greater than baseline numbers in BAL. Antigen-specific T cell responses were measured in the BAL and PBMCs after i.v. BCG. The frequency of memory CD4 T cells in BAL producing IFN-γ, TNF, IL-2, and/or IL-17 after restimulation with Mtb WCL reached a median of 47% by 8 wk and increased 30-fold in number between 2 and 8 wk (Fig. 1, D and E; and Fig. S1 E). Some CD4 T cells in BAL express CD8α after i.v. BCG (DP, Fig. 1 C). When compared with antigen-specific CD4 single-positive (SP) T cells, CD4^+^CD8α^+^ DP cells contain a similarly high frequency of antigen-specific cells (Fig. 1 D) and produce nearly an identical combination of Th1/Th17 cytokines (Fig. 1 F). However, due to their much smaller proportion (Fig. 1 C), CD4^+^CD8α^+^ DP cytokine-producing cells represent a far fewer number of total cells in the BAL. Classical CD8 T cell responses, while not optimally stimulated with WCL, were lower than CD4 T cell responses in both the BAL and PBMCs (Fig. S1, E and F).

### Antibody-mediated depletion was profound in blood and tissues

**Figure 2.**
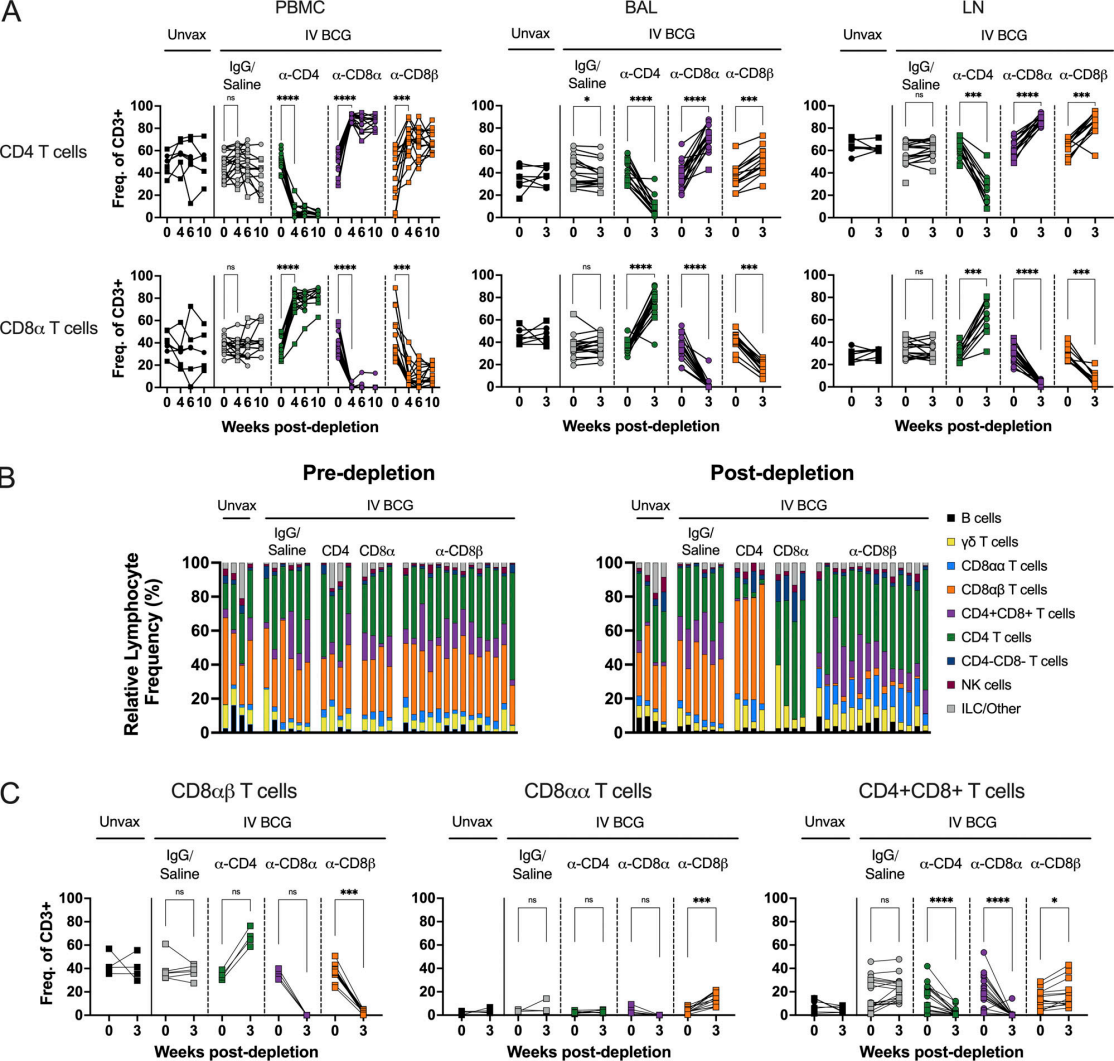
**Target cell populations are depleted by antibody infusions in vivo. (A)** Flow cytometry to assess T cell depletion in blood (PBMCs, left), airway (by BAL, middle), and peripheral LNs (by biopsy, right). For each depletion group, conventional CD4^+^ T cells (CD3^+^CD20^−^γδTCR^−^CD8α^−^) are shown on the top and CD8α^+^ T cells (CD3^+^CD20^−^γδTCR^−^CD4^−^) are shown on the bottom; vaccination status is at the top of the plot and antibody infusion designates depletion group. Each symbol represents an animal, and lines connect each animal across time points. Unvax: *n* = 7; IgG/saline: *n* = 18; α-CD4: *n* = 16; α-CD8α: *n* = 17; and α-CD8β: *n* = 14. **(B)** Population composition in BAL, shown as a relative frequency of lymphocytes. Pre-depletion is shown of the left, post-depletion is shown on the right. Each bar represents an animal. Unvax: *n* = 4; IgG/saline: *n* = 6; α-CD4: *n* = 4; α-CD8α: *n* = 4; and α-CD8β: *n* = 14. Only animals in the second cohort are included, as anti-CD20 and anti-CD8β antibodies were not included in the flow cytometry panels for the first cohort. **(C)** Conventional CD8αβ T cells (left), unconventional CD8αα T cells (middle), and CD4^+^CD8^+^ DP T cells (right) are selectively depleted in the airway (by BAL) following CD8α and CD8β depletion. Populations are reported as a frequency of CD3^+^ T cells. Unvax: *n* = 4–7; IgG/saline: *n* = 6–18; α-CD4: *n* = 4–16; α-CD8α: *n* = 4–17; and α-CD8β: *n* = 14. Only animals in the second cohort are included in CD8αα and CD8αβ cell type analysis, as an anti-CD8β antibody was not included in the flow cytometry panels for the first cohort, limiting statistical comparisons in CD4 and CD8α depletion groups. P value ranges indicated for Wilcoxon matched-pairs signed-rank test comparison of t = 0 and earliest postdepletion time point (i.e., t = 4 for PBMCs, t = 3 for BAL and LN). ns = not significant, P ≥ 0.05; * = 0.01 ≤ P < 0.05; *** = 0.0001 ≤ P < 0.001; and **** = P < 0.0001.

**Figure S2.**
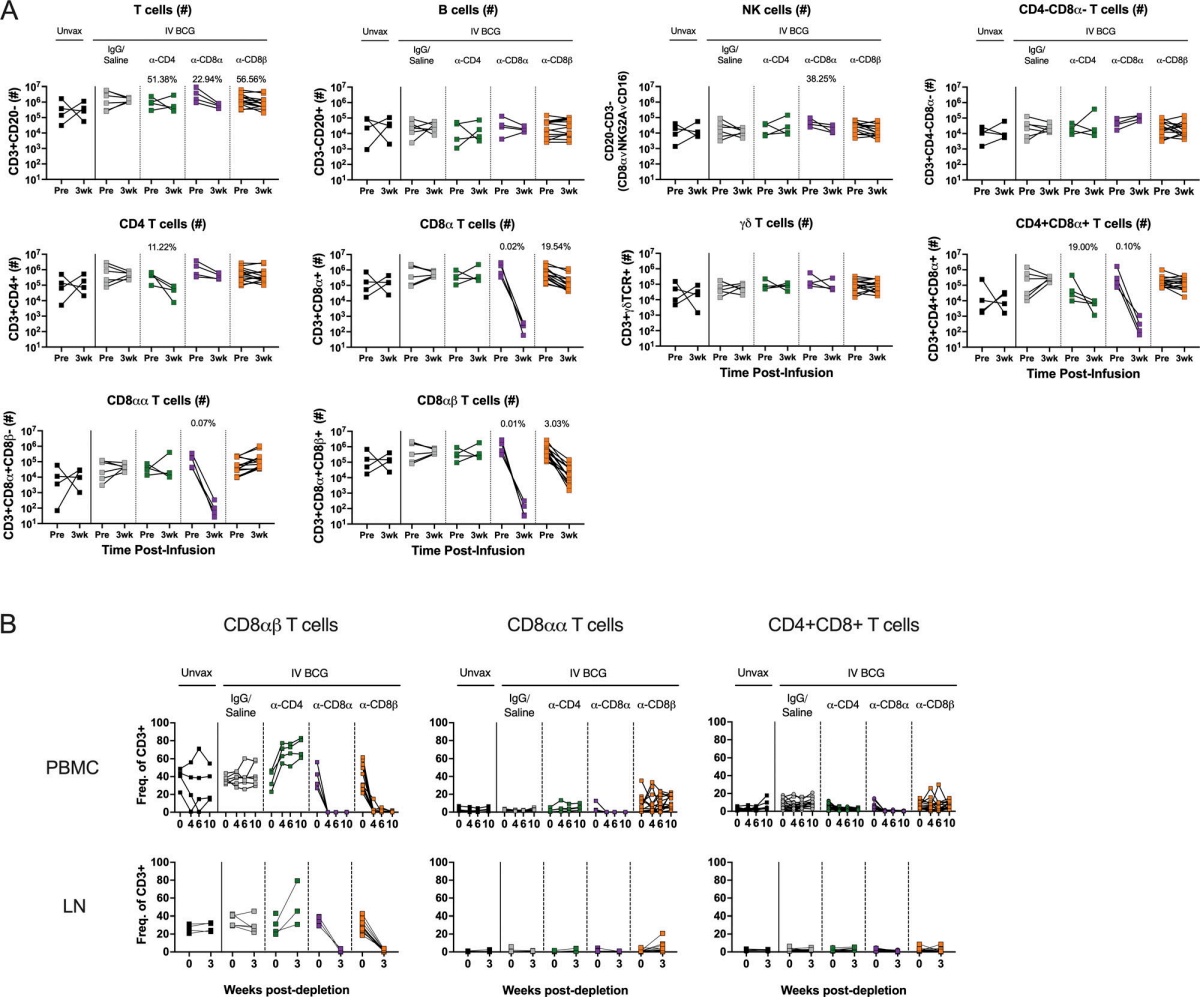
**Targeted lymphocyte subsets were successfully depleted in BAL. (A)** Numbers of all lymphocyte subsets before and after depletion in BAL were characterized by flow cytometry. Only animals in the second cohort are included, as anti-CD20 and anti-CD8β antibodies were not included in the flow cytometry panels for the first cohort. Percentages shown in each plot represent population size relative to pre-depletion samples, calculated by group median. **(B)** CD3^+^CD8^+^ T cell subsets are selectively depleted following CD8α and CD8β depletion. Left panels: conventional CD8αβ T cells (CD20^−^CD3^+^γδTCR^−^CD4^−^CD8α^+^CD8β^+^); middle panels: unconventional CD8αα T cells (CD20^−^CD3^+^γδTCR^−^CD4^−^CD8α^+^CD8β^−^); and right panels: CD4^+^CD8^+^ DP T cells (CD20^−^CD3^+^γδTCR^−^CD4^+^CD8α^+^). Top panels: PBMCs; bottom panels: peripheral LNs. Populations are reported as a frequency of CD3^+^ T cells. Only animals in the second cohort are included for CD8αα and CD8αβ T cell analysis, as an anti-CD8β antibody was not included in the flow cytometry panels for the first cohort. Unvax: *n* = 4–7; IgG/saline: *n* = 6–18; α-CD4: *n* = 4–16; α-CD8α: *n* = 4–17; and α-CD8β: *n* = 14.

Using flow cytometry, we monitored the extent of antibody-mediated depletion of individual lymphocyte populations in the blood (via PBMCs) throughout the depletion phase of the study, as well as in the airways (via BAL) and peripheral LNs (via biopsy) after two infusions. Depletion by each antibody was >90% of the expected populations in PBMCs (Fig. 2 A and Fig. S1). While depletion was most efficient in the blood, results from the airways (CD4: 89% depletion of CD4 T cells, CD8α: 99.9% depletion of all CD8 T cells, and CD8β: 97% depletion of CD8αβ T cells) and LNs (CD4: 81% depletion, CD8α: 99% depletion, and CD8β: 99% depletion) showed that these antibodies were effective even in tissues. The airway, where Mtb first interacts with the host, had a fundamentally altered cellular profile in each depletion group (Fig. 2 B). These data align with more extensive depletion analyses performed in i.v. BCG–vaccinated rhesus macaques that were not challenged with Mtb (Sutton et al., 2024). As expected, a substantial reduction in CD4^+^ T cells was only observed in each tissue compartment in the anti-CD4 group. The increase in the frequency of CD4s seen in both CD8 depletion groups is a result of the missing subsets from the CD3^+^ population and is not indicative of an increase in cell numbers (Fig. 2, A and B; and Fig. S2). CD8α^+^ T cells, including both CD8αα and CD8αβ cells in the CD3^+^γδ TCR^−^ population, were depleted in both anti-CD8α and anti-CD8β groups. Distinguishing targeted effects in each of these populations required a more detailed analysis of the CD3^+^CD8α^+^ populations (Fig. 2 C and Fig. S2 B). Classical adaptive CD8 T cells (i.e., CD8αβ T cells) were depleted in the airway by both anti-CD8 antibodies. Populations that express the CD8αα homodimer were only depleted by anti-CD8α and not anti-CD8β antibody.

In macaques, it is not feasible to target singular cell types without depletion effects in other lymphocyte subsets. For example, CD4^+^CD8α^+^ DP T cells were reduced in both the anti-CD4 and anti-CD8α groups (Fig. 2 C and Fig. S2). While the exact classification of these cells is debated between a distinct T cell subset and an activated CD4 phenotype, their absence across both depletion groups is important for contextualizing disease outcomes of the two experimental groups.

**Figure 3.**
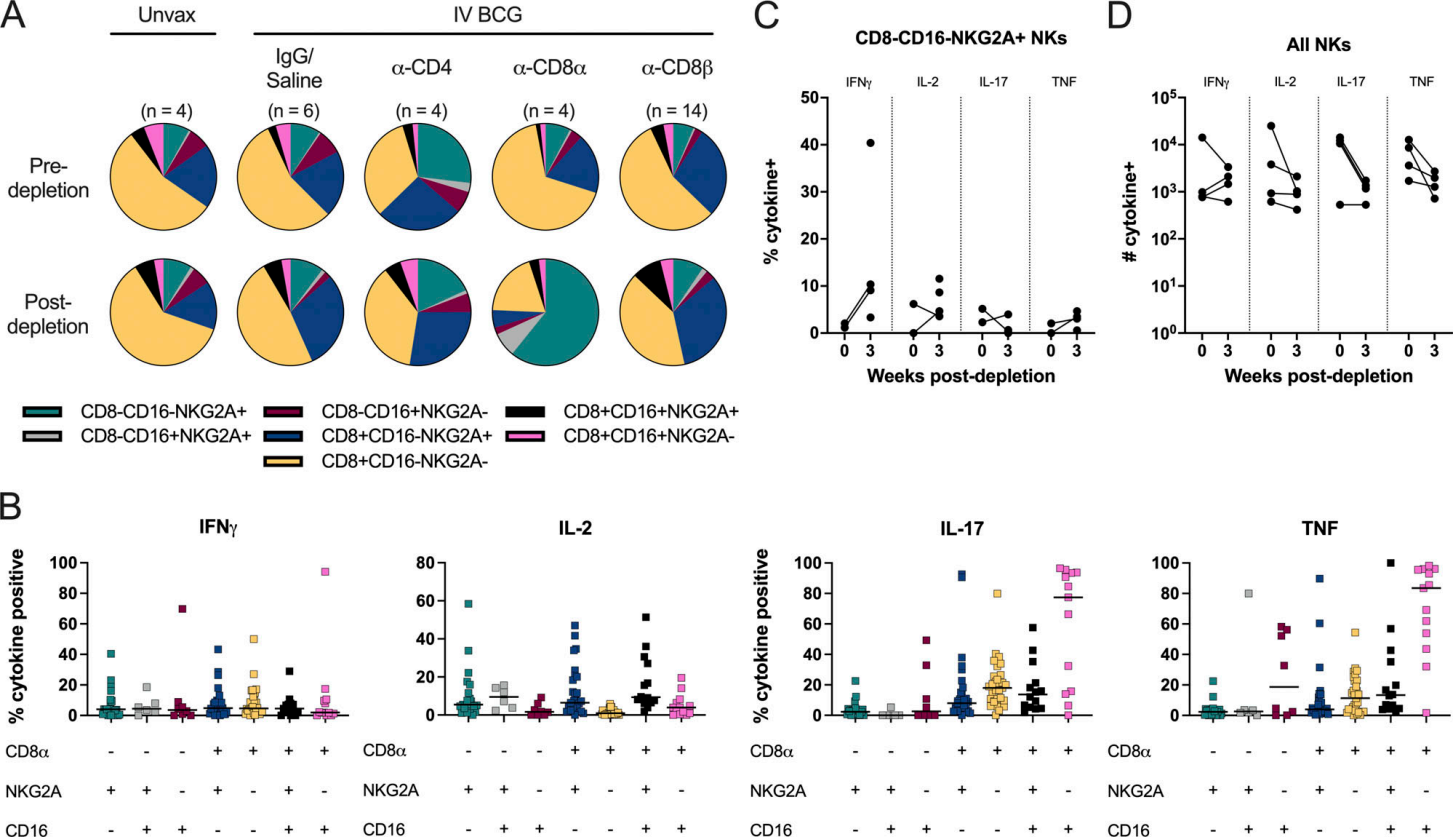
**CD8α depletion shifts phenotypic and functional profile of NK cells in the airway. (A)** Relative frequency of NK (CD3 negative) subtypes in BAL before (top) and after depletion (bottom). Unvax: *n* = 4; IgG/saline: *n* = 6; α-CD4: *n* = 4; α-CD8α: *n* = 4; and α-CD8β: *n* = 14. **(B)** Cytokine (IFN-γ, IL-2, IL-17, and TNF) production by NK subtype using CD8α, NKG2A, and CD16 markers in BAL of animals before depletion, represented as the frequency of cytokine-positive NK cells by flow cytometry (*n* = 65). Each symbol represents an animal, and lines represent group median. If subtype was below event count threshold by flow cytometry that animal will not appear as a symbol for that specific cell type. Subtypes are same colors in A and B. **(C)** Frequency of cytokine-positive NKG2A SP NK cells (CD8α^−^CD16^−^) before and after CD8α depletion. **(D)** Number of NK cells expressing IFN-γ, IL-2, IL-17, or TNF before and after CD8α depletion. Each symbol in C and D represents an animal (*n* = 4).

There is no clear consensus on an exact definition of NK cells in NHPs, including methods of distinguishing them from other innate lymphoid cell (ILC) populations. Further complicating the evaluation of these subsets is the fact that NK cells in blood and tissues are phenotypically heterogeneous (Diedrich et al., 2023; Hong et al., 2013; Sojka et al., 2014). An inclusive definition of NK cells as CD3^−^CD20^−^ lymphocytes expressing CD8α, NKG2A, or CD16 expression was used across tissues. This could include ILCs, but it ensures a complete analysis of a potentially key cell type. Depletion of NK cells with the anti-CD8α antibody was not completely effective (Fig. S2). NK cells were characterized into different subsets based on expression of CD8α, NKG2A, or CD16. Pre-depletion, the NK population in the airway was dominated by CD8α^+^NKG2A^−^CD16^−^ and CD8α^+^NKG2A^+^CD16^−^ subsets (Fig. 3 A). Intracellular cytokine staining of BAL from vaccinated but undepleted animals by flow cytometry showed that CD8α^+^ subsets produce high levels of IL-17 and TNF, while NKG2A^+^ subsets were associated with elevated IL-2 (Fig. 3 B). Following CD8α depletion, CD8α^+^ NKs were largely replaced by NKG2A (CD159a) SP cells (Fig. 3 A), but the functional profile of NKG2A^+^ SP cells did not change to compensate for lost cytokine production, with the potential exception of more IFN-γ (Fig. 3 C). This led to a significant shift in the overall NK cell functional profile, as the number of IL-17 and TNF-expressing NK cells in the airways dropped by roughly 10-fold, a ∼90% decrease (Fig. 3 D).

### CD4 and CD8α^+^ lymphocytes are necessary for i.v. BCG–induced protection

**Figure 4.**
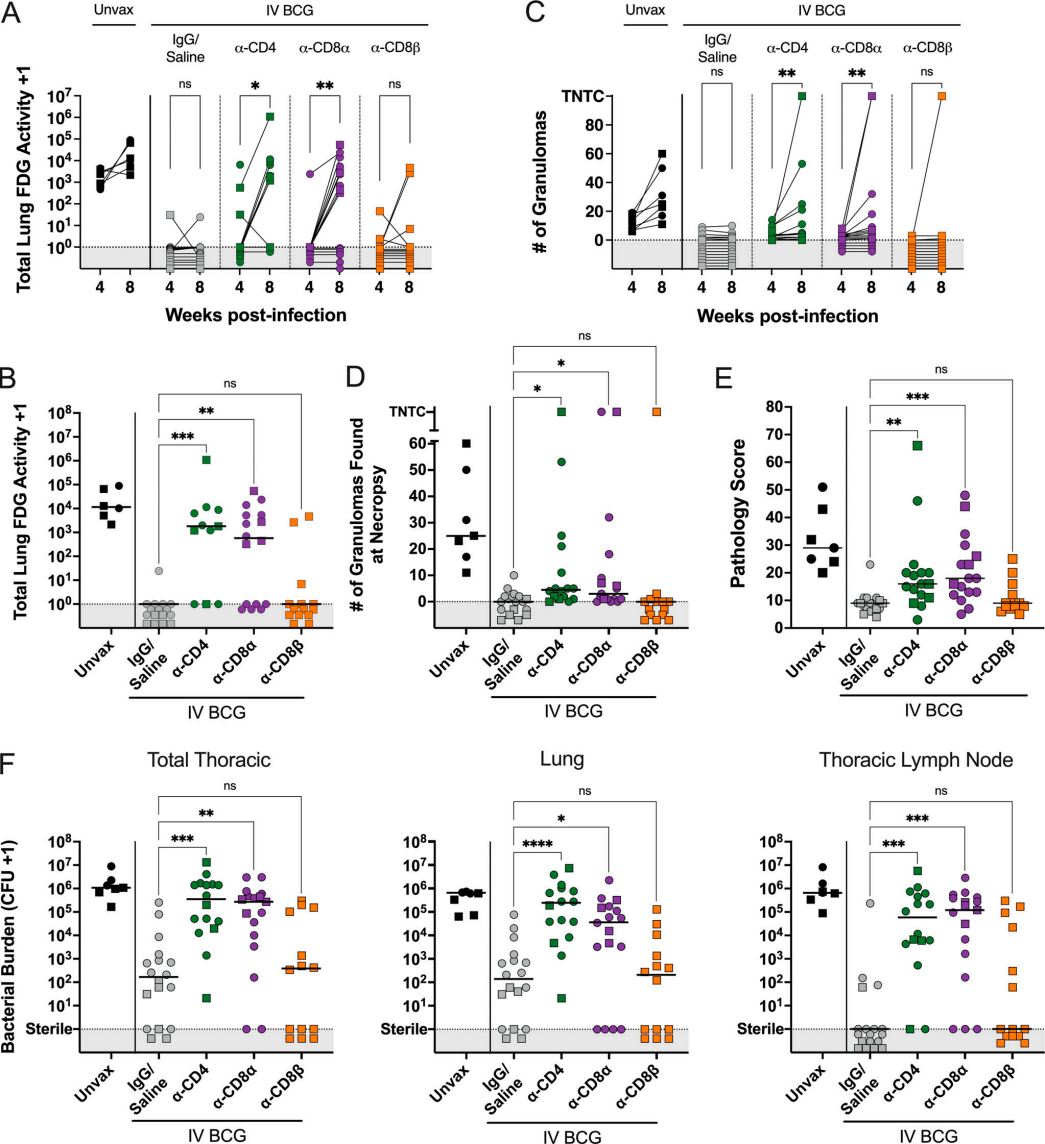
**CD4 and CD8α depletion lead to increased disease and bacterial burden. (A)** Total lung FDG activity at 4 and 8 wk after Mtb challenge. Animals with missing PET scans were not included. Unvax: *n* = 7 (4 wk), 6 (8 wk); IgG/saline: *n* = 16 (4 and 8 wk); α-CD4: *n* = 15 (4 wk), 11 (8 wk); α-CD8α: *n* = 16 (4 and 8 wk); and α-CD8β: *n* = 14 (4 and 8 wk). **(B)** Total lung FDG activity at necropsy. Animals with missing PET scans were not included. Unvax: *n* = 6; IgG/saline: *n* = 16; α-CD4: *n* = 11; α-CD8α: *n* = 16; and α-CD8β: *n* = 14. **(C)** Number of granulomas seen by PET CT scans at 4 and 8 wk after infection. TB pneumonia or consolidations are denoted as too numerous to count (TNTC). For A and C, each symbol represents an animal, and lines connect animals across time points. P value ranges indicated for Wilcoxon matched-pairs signed-rank test comparison of 4 versus 8 wk after Mtb challenge. **(D)** The number of granulomas found at necropsy. TB pneumonia and consolidations are represented as TNTC. **(E)** Gross pathology score, as described by Maiello et al. (2018). **(F)** Bacterial burden (CFUs) of all thoracic tissues (left), lung (middle), and thoracic LNs (right). Symbols represent an animal. For B and D–F: Unvax: *n* = 7; IgG/saline: *n* = 18; α-CD4: *n* = 16; α-CD8α: *n* = 17; and α-CD8β: *n* = 14. Circles represent cohort 1 and squares represent cohort 2. In all panels except E, symbols in gray regions are of equal value (0 or sterile) and were spread for better visualization. Groups were compared using the Kruskal–Wallis test, with Dunn’s multiple comparison adjusted P value ranges indicated, comparing IgG/saline group against each depletion group. ns = not significant P ≥ 0.05; * = 0.01 ≤ P < 0.05; ** = 0.001 ≤ P < 0.01; *** = 0.0001 ≤ P < 0.001; and **** = P < 0.0001.

Serial PET CT scanning was performed to monitor Mtb infection trajectory and disease over time (Fig. 4 A). The positive control, undepleted (IgG/saline group) i.v. BCG–vaccinated animals had lower total lung FDG activity (inflammation) compared with unvaccinated animals, consistent with previous studies (Darrah et al., 2020, 2023). While the depleted groups showed minimal inflammation at 4 wk after infection, most CD4- and CD8α-depleted macaques had high lung FDG activity by 8 wk after Mtb, with significantly higher total lung FDG activity compared with IgG/saline-vaccinated animals (Fig. 4 B). In contrast, CD8β-depleted vaccinated animals were similar to undepleted, vaccinated animals in total lung FDG activity. The majority of the CD8β depletion group did not show increased lung FDG activity, with only 2 NHPs having high PET signal in the lungs at 8 wk after infection, in contrast to 9 of 15 in the CD8α depletion group.

The number of granulomas identified on PET CT scans 4 wk after infection indicated that vaccinated animals formed relatively few lesions at 4 wk after infection, regardless of depletion (0–14 granulomas in vaccinated animals with or without depletion compared with 6–19 granulomas in unvaccinated animals) (Fig. 4 C). By 8 wk after infection, granulomas increased in all animals in the unvaccinated group and in a subset of animals in the CD4- and CD8α-depleted groups. This was not observed in the i.v. BCG animals that received IgG/saline or anti-CD8β antibody infusions.

Detailed necropsies were performed using the final PET CT scan as a map of disease. Unexpectedly, the numbers of granulomas grossly identified at necropsy in many of the depleted vaccinated macaques were lower than unvaccinated animals (median: 25) (Fig. 4 D). Still, the CD4-depleted group (median: 4.5) and CD8α-depleted group (median: 3) had significantly higher numbers of granulomas than the nondepleted vaccinated controls (median: 0), and a small number of clearly unprotected animals in each group had extensive disease (e.g., TB pneumonia). CD8β-depleted animals had very few granulomas at necropsy, similar to undepleted animals (median: 0). Gross pathology was scored by evaluating the number and size of granulomas and other lung pathologies, size and granuloma involvement of LNs, and evidence of extrapulmonary (EP) dissemination (Lin et al., 2009). As seen previously, i.v. BCG vaccination reduced the gross pathology score compared with unvaccinated animals (median: 9 versus 29; Fig. 4 E) (Darrah et al., 2020). Both anti-CD4–depleted (median: 16) and anti-CD8α–depleted (median: 18) macaques had significantly higher gross pathology scores compared with undepleted, vaccinated controls. In contrast, CD8β-depleted animals were not significantly different from IgG/saline-treated vaccinated controls.

Multiple individual tissue homogenates (all lung granulomas and other pathologies, multiple random samples of each lung lobe, and all thoracic LNs) were plated and counted to calculate total thoracic bacterial burden as Mtb CFUs. Peripheral LNs, spleen, and liver were also plated. Mtb sequencing analysis, discussed further below, confirmed that the colonies recovered were Mtb and not BCG. i.v. BCG vaccination resulted in a ∼10,000-fold reduction in bacterial burden compared with unvaccinated controls (median i.v. BCG: 166 CFUs versus median unvaccinated 1.08 × 10^6^ CFUs) (Fig. 4 F), consistent with previous studies (Darrah et al., 2020, 2023). Despite the small number of lesions observed by PET CT or recovered during necropsy of depleted NHPs, plating tissues for CFU revealed substantial thoracic bacterial burden in both CD4-depleted (median: 3.5 × 10^5^ CFUs) and CD8α-depleted (median: 2.7 × 10^5^ CFUs) groups, which were significantly higher compared with the vaccinated controls and similar to unvaccinated animals. CD8β-depleted animals were similar to undepleted, vaccinated controls in bacterial burden with several sterile (no Mtb recovered) macaques. In striking contrast, no CD4-depleted NHPs (0/16) showed sterilizing immunity, although one animal had only 20 total CFUs, so would still be considered to be protected (<100 CFUs). Two CD8α depleted animals (2/17) had no Mtb recovered from any sites. CD4- and CD8α-depleted animals were significantly higher than vaccinated controls in both lung CFU and thoracic LN CFU.

**Figure 5.**
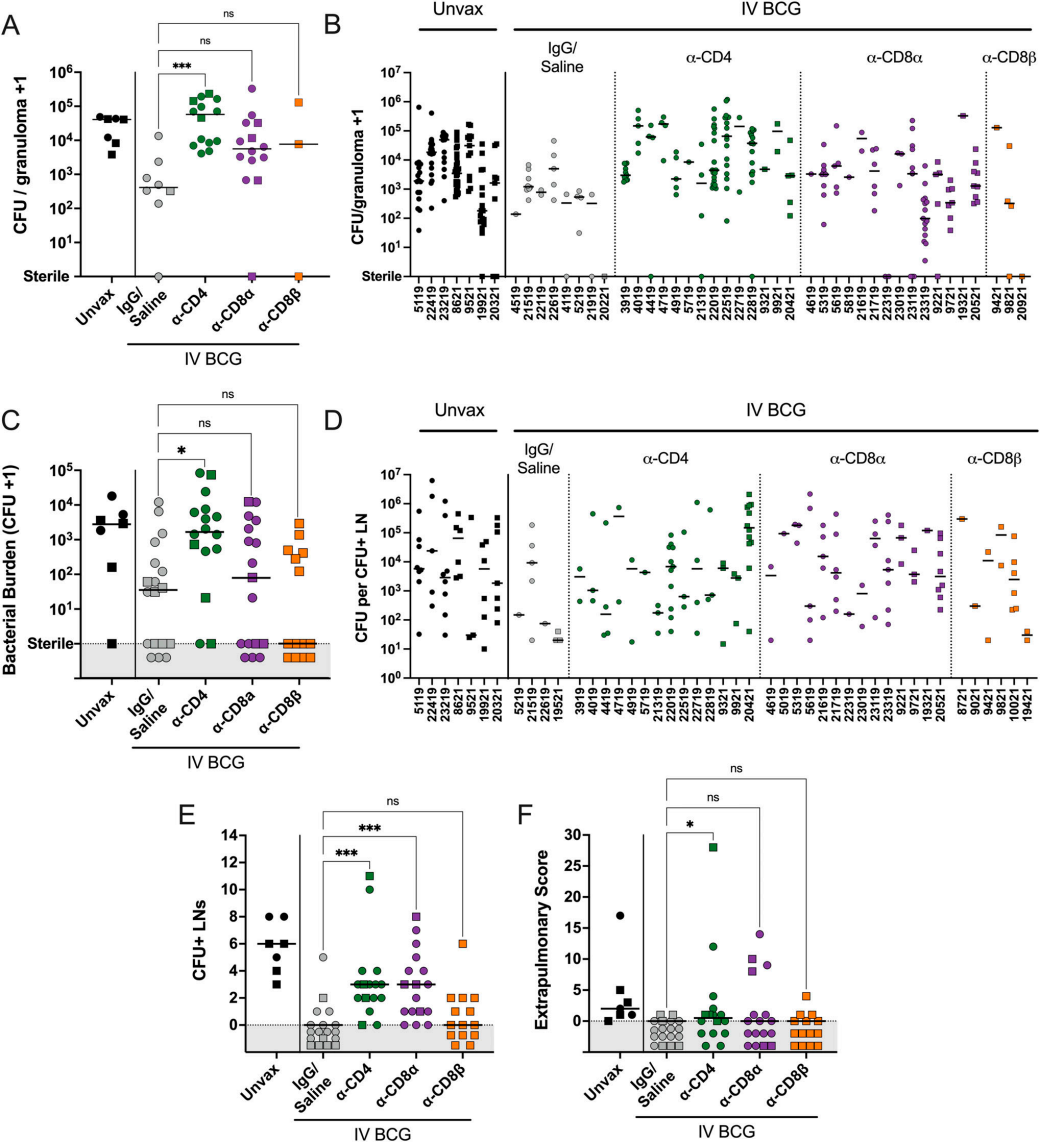
**Granulomas and dissemination events are less controlled following CD4 and CD8α depletion. (A)** The bacterial burden per granuloma. Symbols represent a mean of all granulomas within an animal, and animals with no granulomas are not included. **(B)** CFUs per granuloma separated by animal. **(C)** Bacterial burden in lung lobes without gross pathology (i.e., non-granuloma tissue). **(D)** CFUs of non-sterile thoracic LN separated by animal. In B and D, each symbol represents a granuloma or LN, and each column represents an animal. **(E)** The number of CFU^+^ thoracic LNs per animal. **(F)** The EP score, as described by Maiello et al. (2018). For all panels, each symbol represents an animal, line represents group median; circles represent cohort 1 and squares represent cohort 2. In C–F, symbols in gray regions are of equal value (0 or sterile) and were spread for better visualization. All symbols in gray region are of equal value (0 or sterile) and were spread for better visualization. Unvax: *n* = 7; IgG/saline: *n* = 18; α-CD4: *n* = 16; α-CD8α: *n* = 17; and α-CD8β: *n* = 14. Groups were compared using the Kruskal–Wallis test, with Dunn’s multiple comparison adjusted P value ranges indicated, comparing IgG/saline group against each depletion group. ns = not significant, P ≥ 0.05; * = 0.01 ≤ P < 0.05; and *** = 0.0001 ≤ P < 0.001.

Previously, our data supported that there is a limit to the bacterial capacity of granulomas before failure and dissemination occur (Lin et al., 2014). i.v. BCG vaccination reduced the number of bacteria recovered from granulomas (median undepleted i.v. BCG: 411 CFUs versus median unvaccinated: 4.1 × 10^4^ CFUs), indicating effective early control. A significant increase in CFUs per granuloma was observed in the CD4 depletion group (median: 5.8 × 10^4^ CFUs) (Fig. 5 A). Most granulomas in CD4-depleted animals contained higher bacterial burden than those from undepleted animals, implicating CD4 T cells in limiting bacterial growth within granulomas (Fig. 5 B). There was a trend toward increased bacterial burden in granulomas from CD8α-depleted animals (P = 0.1494) (Fig. 5 A). The low number (*n* = 2) of CD8β-depleted animals that formed granulomas precludes meaningful conclusions being drawn for that group.

### High Mtb burden in depleted lung tissue leads to increased effector molecule production

**Figure 6.**
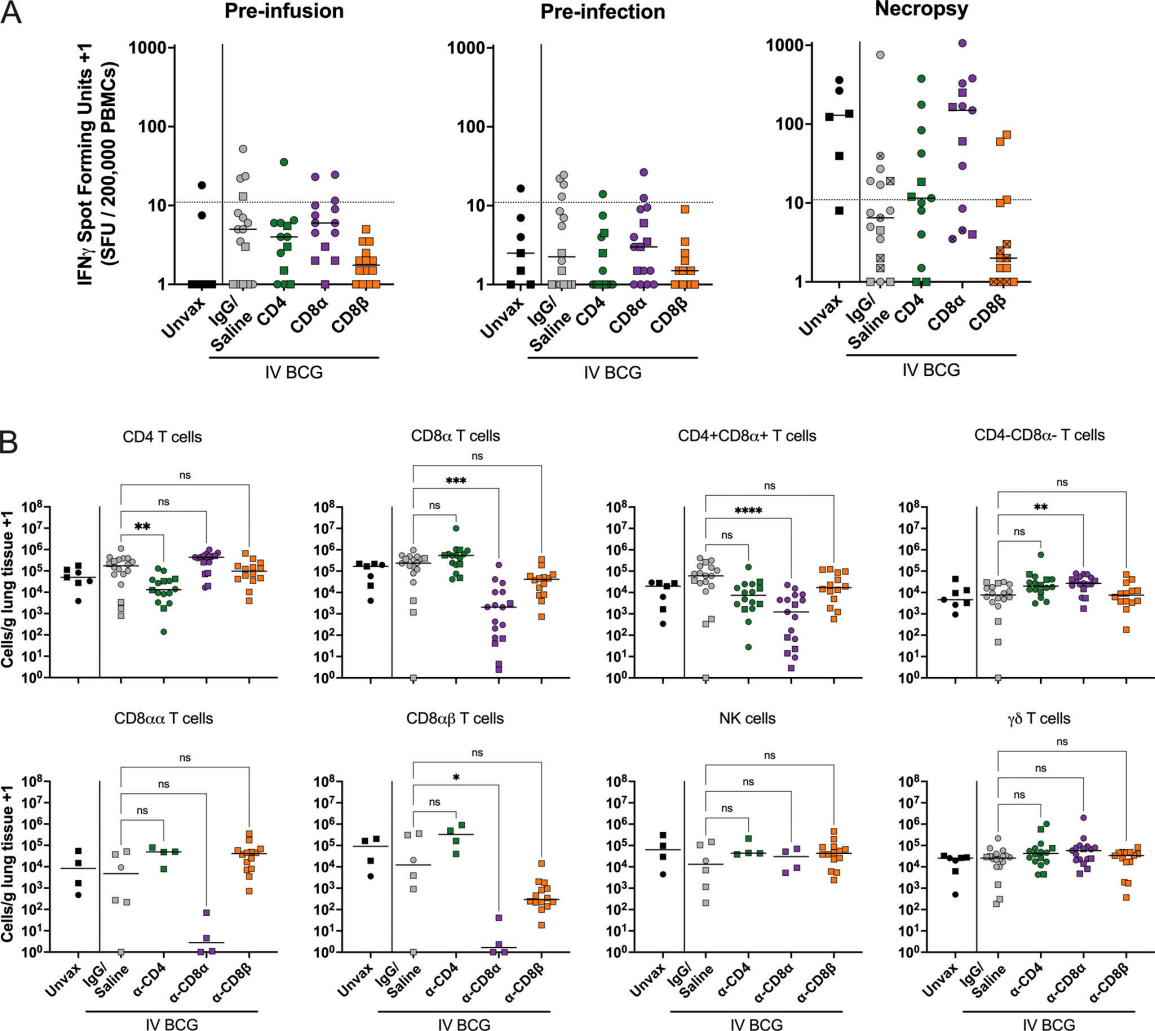
**Mtb-specific systemic responses and immune profile in lungs are altered after depletion. (A)** ELISpot IGRA results following stimulation with ESAT6 and CFP10 peptide pools. SFUs were normalized to unstimulated background and averaged across two replicates. A response of 10 SFUs per 200,000 PBMCs is considered positive for an Mtb-specific response (Darrah et al., 2020). Pre-infusion: post-vaccination, pre-depletion (Unvax: *n* = 6; IgG/saline: *n* = 16; α-CD4: *n* = 13; α-CD8α: *n* = 14; and α-CD8β: *n* = 14). Pre-infection: post-depletion, pre-Mtb challenge (Unvax: *n* = 7; IgG/saline: *n* = 16; α-CD4: *n* = 14; α-CD8α: *n* = 16; and α-CD8β: *n* = 14). Necropsy: at necropsy (Unvax: *n* = 6; IgG/saline: *n* = 17; α-CD4: *n* = 13; α-CD8α: *n* = 13; and α-CD8β: *n* = 14). Lines represent group median. Symbols with a cross represent sterile animals. **(B)** Number of each lymphocyte subset per gram of lung tissue, characterized by flow cytometry. Each symbol represents an animal; circles represent cohort 1 and squares represent cohort 2. Unvax: *n* = 7; IgG/saline: *n* = 18; α-CD4: *n* = 16; α-CD8α: *n* = 17; and α-CD8β: *n* = 14. Percentages shown in each plot represent population size relative to IgG/saline group, calculated by group median (line). Only cohort 2 was included for CD8αα, CD8αβ, and NK cell quantification, as anti-CD20 and anti-CD8β antibodies were not included in the flow cytometry panels in cohort 1 (Unvax: *n* = 4; IgG/saline: *n* = 6; α-CD4: *n* = 16; α-CD8α: *n* = 17; and α-CD8β: *n* = 14). Each symbol represents a mean per animal of two to four lung lobes sampled, bar represents group median. Groups were compared using the Kruskal–Wallis test, with Dunn’s multiple comparison adjusted P value ranges indicated (ns = not significant, P ≥ 0.05; * = 0.01 ≤ P < 0.05; ** = 0.001 ≤ P < 0.01; *** = 0.0001 ≤ P < 0.001; and **** = P < 0.0001), comparing IgG/saline group against each depletion group.

IFN-γ release assays (IGRAs) are a key component in diagnosing Mtb infection. We used IFN-γ enzyme-linked immunospot (ELISpots) to assess Mtb-specific responses in blood following Mtb challenge using early secreted antigenic target 6 kDA (ESAT-6) and culture filtrate protein 10 (CFP10) peptide pools as stimulators. ESAT-6 and CFP10 are not expressed by BCG due to its attenuation, enabling a differentiation of vaccine- and infection-induced responses. Five of six unvaccinated macaques showed a positive ELISpot result (>10 spot-forming units [SFUs]/200,000 PBMCs) at necropsy (Fig. 6 A). In the undepleted, vaccinated group, 11 of 17 of the animals had negative Mtb-specific responses, as in our previous study (Darrah et al., 2020). This also held true in the CD8β depletion group, with 12/14 animals returning negative results. These animals had not developed significant disease and likely controlled infection effectively before an adaptive T cell response specific to Mtb could be formed. Approximately half (7/13) of CD4-depleted macaques and most (9/13) CD8α-depleted macaques had positive ESAT6/CFP10 ELISpots at necropsy. While the proportion of CD4- and CD8α-depleted animals testing positive was similar, the amplitude of response was lower in the CD4-depleted group (median 10.5 SFUs/200,000 cells) than the CD8α-depleted group (median 148.5 SFUs/200,000 cells). These assays are largely driven by CD4 T cell IFN-γ production, so it is reasonable to expect stunted responses after CD4 depletion despite high bacterial burden.

**Figure S3.**
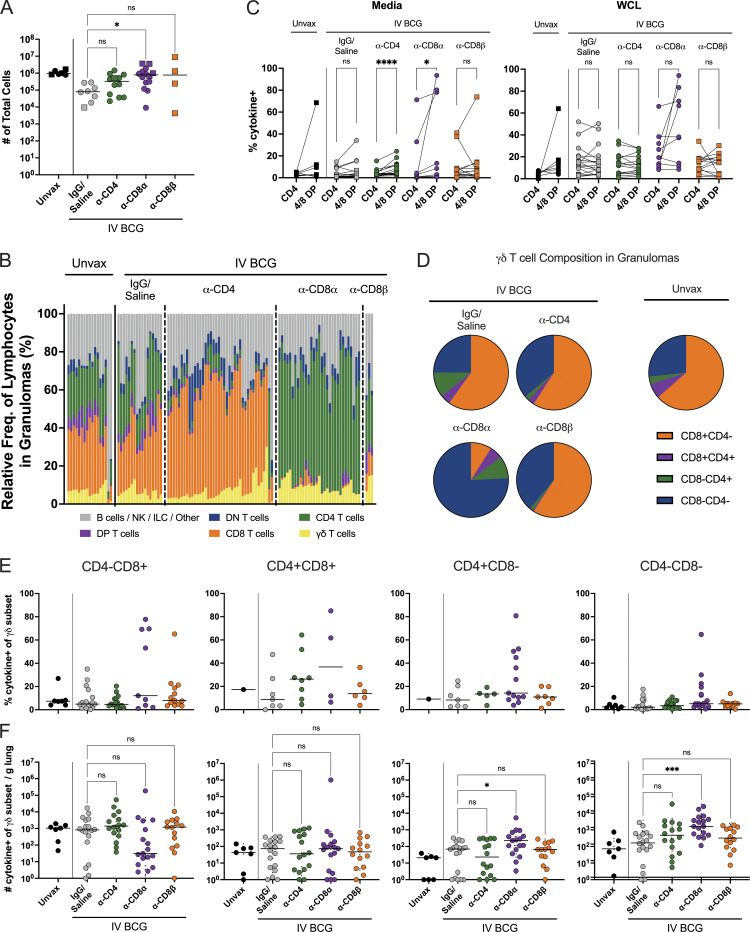
**Granuloma lymphocyte composition. (A)** Total number of cells per granuloma. Each symbol represents the mean of all granulomas in an animal, line represents the group median. All groups (excluding the unvaccinated animals) were compared using the Kruskal–Wallis test with Dunn’s multiple comparison adjusted P value ranges indicated, comparing IgG/saline group against each depletion group. Circles represent cohort 1 and squares represent cohort 2. **(B)** The relative frequencies of lymphocyte populations in granulomas. Each bar represents a granuloma, divided by depletion group. B/NK/ILC/other are CD3^−^ populations. **(C)** Cytokine production by CD4^+^CD8^−^ SP and CD4^+^CD8^+^ DP T cells incubated without stimulation (Media, left) or with WLC (right). P value ranges indicated for Wilcoxon matched-pairs signed-rank test comparison of CD4 SP and CD4^+^CD8α^+^ DP T cells under each stimulation condition. **(D)** Relative abundance of CD4^+^ and/or CD8α^+^ γδ T cells found in granulomas. Animals with no granulomas were not included in A, B, and D. Unvax: *n* = 7; IgG/saline: *n* = 8; α-CD4: *n* = 12; α-CD8α: *n* = 14; and α-CD8β: *n* = 4. **(E and F)** Production of IFN-γ, TNF, IL-2, and/or IL-17 by each gd T cell subset as a frequency of cells in that subset (E) and number (F). Groups in F were compared as in A. For C, E, and F: Unvax: *n* = 7; IgG/saline: *n* = 18; α-CD4: *n* = 16; α-CD8α: *n* = 17; and α-CD8β: *n* = 14. ns = not significant, P ≥ 0.05; * = 0.01 ≤ P < 0.05; *** = 0.0001 ≤ P < 0.001; and **** = P < 0.0001.

While IGRAs are a useful clinical tool, granulomas in the lung are the primary battleground between the immune system and the invading pathogen. To determine how these cell populations may be influencing infection dynamics, flow cytometry was used to analyze the populations present within these tissues and their functionality. i.v. BCG–vaccinated, undepleted animals generally have fewer cells collected from granulomas (Fig. S3 A), likely because they controlled the infection early and are healing by the time of excision at necropsy. The number of cells in granulomas did not change following CD4 or CD8β depletion but significantly increased following depletion of all CD8α^+^ lymphocytes. Relative frequencies of lymphocyte subsets in excised granulomas were consistent between unvaccinated and undepleted, vaccinated groups (Fig. S3 B). Populations not targeted by depletion (e.g., CD4 T cells in CD8α depletion) were found in higher frequencies within lesions.

CD4^+^CD8α^+^ and γδ T cells are cell types lost following both CD4 and CD8α depletion that might be playing an important role in restricting Mtb infection at the granuloma. We hypothesized that CD4^+^CD8^+^ DP T cells could be playing a role in i.v. BCG–induced protection since this population was at least partially depleted in both groups. The DP population has previously been shown to be enriched for antigen-specific responses in chronic cytomegalovirus infection of rhesus macaques (Macchia et al., 2006). However, while this phenomenon was observed in naïve, unvaccinated animals, it did not persist in rhesus macaques after i.v. BCG. Similarly to the airway following vaccination, DP cells in lung tissue after Mtb challenge contribute to the overall response, but are not enriched for antigen positivity when compared with CD4 SP T cells (Fig. S3 C). Analysis of this population in lung tissue at necropsy indicated that, while there is a trend toward DP CD4 T cells producing more cytokines than SP CD4 T cells without ex vivo antigen stimulation (P = 0.1205), negligible differences in function were seen between single- and DP CD4 T cells following stimulation with WCL (P = 0.6112; Fig. S3 C). In fact, functional CD4^+^CD8α^+^ T cells remained following CD8α depletion, although in small numbers (Fig. 6 B). This suggests that CD4^+^CD8α^+^ T cells are highly activated following vaccination and may, therefore, contribute to rapid control of Mtb infection in some manner, but SP CD4 T cells are also activated and antigen specific with similar functions to DP CD4 T cells.

In lung granulomas of naïve macaques or those vaccinated with i.v. BCG (undepleted), ∼60% of γδ T cells are CD4^−^CD8α^+^ and ∼25% are CD4^−^CD8α^−^ (Fig. S3 D) (Kalyan and Kabelitz, 2013). The most prevalent subtype, CD4^−^CD8α^+^ γδ T cells, was efficiently depleted by anti-CD8α antibody. However, the total number of γδ T cells in lung tissue was unchanged by depletion (Fig. 6 B). Instead, the proportion of CD8α^−^ γδ T cells in granulomas increased and had similar function as CD8α^+^ γδ T cells, thereby replacing production of IFN-γ, TNF, IL-2, and/or IL-17 (Fig. S3, D and E). Thus, the overall γδ T cell population remained constant and maintained function even upon depletion via an influx of CD4^−^CD8α^−^ DN γδ T cells (Fig. S3 F).

**Figure 7.**
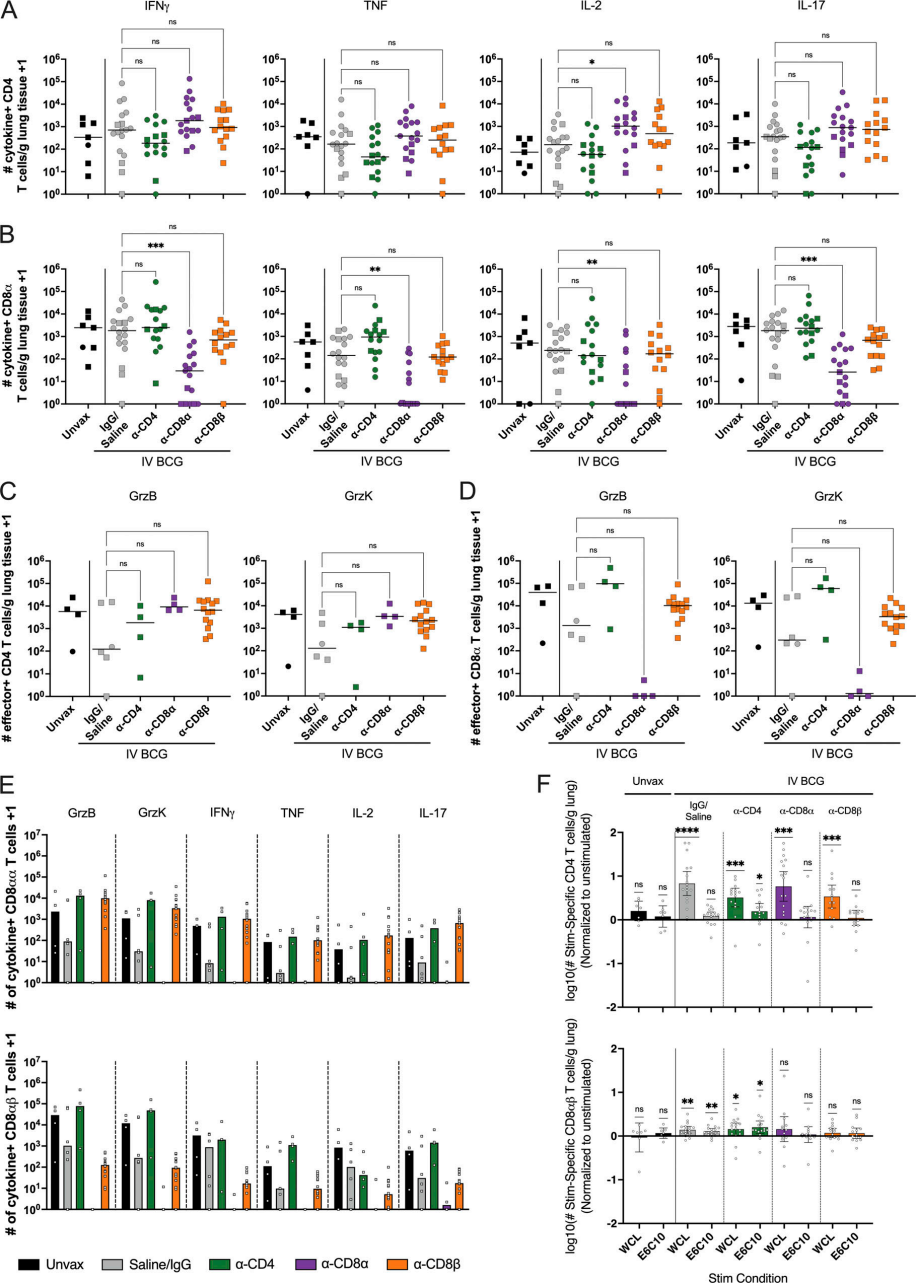
**Functional lymphocytes are depleted in lung tissue. (A and B)** Number of CD4 (A) and CD8α^+^ (B) T cells per gram of lung tissue producing IFN-γ, TNF, IL-2, or IL-17. **(C and D)** Number of CD4 (C) and CD8α^+^ (D) T cells per gram of lung tissue producing granzyme B or granzyme K. Groups were compared using the Kruskal–Wallis test, with Dunn’s multiple comparison adjusted P value ranges indicated, comparing IgG/saline group against each depletion group. **(E)** CD8α^+^ T cell responses categorized by CD8αα (top) and CD8αβ (bottom) T cells as subsets of the CD8α T cells in B. **(F)** Number of CD4 (top) and CD8αβ^+^ (bottom) T cells producing cytokines (IFN-γ/TNF/IL-2/IL-17) when stimulated with WCL (W) or ESAT-6 and CFP10 peptide pools (E6C10) compared with baseline production without stimulation. P value ranges indicated for one-sample *t* test. For E and F, each symbol represents a mean per animal of two to four lung lobes sampled, bar represents group median. Granzymes were only analyzed in cohort 2. Lines connect animals. Unvax: *n* = 7; IgG/saline: *n* = 18; α-CD4: *n* = 16; α-CD8α: *n* = 17; and α-CD8β: *n* = 14. Symbols represent an animal, circles represent cohort 1, and squares represent cohort 2. ns = not significant, P ≥ 0.05; * = 0.01 ≤ P < 0.05; ** = 0.001 ≤ P < 0.01; *** = 0.0001 ≤ P < 0.001; and **** = P < 0.0001.

Successful depletion was also observed in grossly uninvolved lung tissue, where we were able to sample larger amounts of tissue and therefore recover more cells than from individual granulomas (Fig. 6 B). Despite depletion of >90% of CD4 T cells in the lung, there remained a small number of cytokine-positive CD4 T cells detected by flow cytometry (Fig. 7 A). There was a trend (P = 0.0704) toward more CD4 T cells producing IL-2 in the CD8α depletion group. This may be an indication of the immune system trying to compensate for the perturbation, as IL-2 can elicit cytotoxic effector functions from CD8 T and NK cells. CD8α depletion was nearly complete in its elimination of cytokine-producing CD8^+^ cells (Fig. 7 B). It appears as though the population not targeted by the depletion antibody (e.g., CD4 T cells following CD8α depletion) provides some level of compensatory cytokine production, again likely due to high bacterial burden rather than a phenotypic change; however, our study was not powered to evaluate such subtle differences. Similar trends were seen in cytotoxic effector production (Fig. 7, C and D). As it has been demonstrated previously that effector/memory populations are effectively depleted in tissue by antibody infusions, it is unlikely that this represents a change in functional profile of the cell types, such as CD4 T cells adopting a cytotoxic phenotype in the absence of CD8α^+^ cells (Sutton et al., 2024; Winchell et al., 2023). Rather, it is likely the result of heightened disease burden in depleted animals leading to increased activation of those remaining CD4 T cells. In the CD8β-depleted lung tissue, the remaining functional CD8α^+^ T cells are primarily CD8αα (Fig. 7 E). Ex vivo stimulation with WCL showed vaccine-induced memory responses, especially in CD4 T cells (Fig. 7 F). However, while significant in undepleted and α-CD4 groups, antigen-specific CD8αβ T cell responses were less abundant than CD4 T cell responses. This could be, in part, a result of poor stimulation of these cells by WCL. Stimulation with a peptide pool of ESAT-6 and CFP10 showed negligible Mtb-specific responses in either CD4 or CD8α T cells regardless of depletion, despite the increase in disease observed in depleted animals. 8 wk may be too early to see significant tissue resident Mtb antigen-specific populations, which are slow to develop in TB, illustrated by a lack of Mtb-specific functionality in the unvaccinated group. Further, any responses, whether to WCL or ESAT-6 and CFP10, may be masked by in vivo stimulation from residual antigen in the lung tissue (i.e., effector molecule production without ex vivo stimulation).

### CD4 and CD8α depletion leads to increased Mtb dissemination

Dissemination, or spread of Mtb infection, is a useful indicator of a lack of immune containment. In CD4-depleted animals, grossly uninvolved lung tissue had 48-fold more CFUs than undepleted, vaccinated controls, indicating dissemination through the lung tissue (Fig. 4 C); CD8α-depleted animals had 2.25-fold more CFUs in uninvolved lung tissue compared with undepleted, vaccinated controls. Thoracic LNs are common destinations for dissemination events, illustrated by unvaccinated animals having a median of six involved (Mtb-positive) thoracic LNs (Fig. 4, D and E). Since infection is generally controlled early in the lung following i.v. BCG precluding dissemination, there was little spread of Mtb from lung to thoracic LNs in undepleted, vaccinated controls. We observed significant increases in the number of CFU^+^ LNs following both CD4 and CD8α depletions, implicating these lymphocytes in limiting disease progression. Despite significant impact on dissemination to LNs, depletion did not greatly increase EP dissemination (Fig. 4 F). However, there was a small but statistically significant increase in EP dissemination following CD4 depletion; it is possible that other depletion groups would show enhanced dissemination if the planned time point for necropsy was extended.

**Figure 8.**
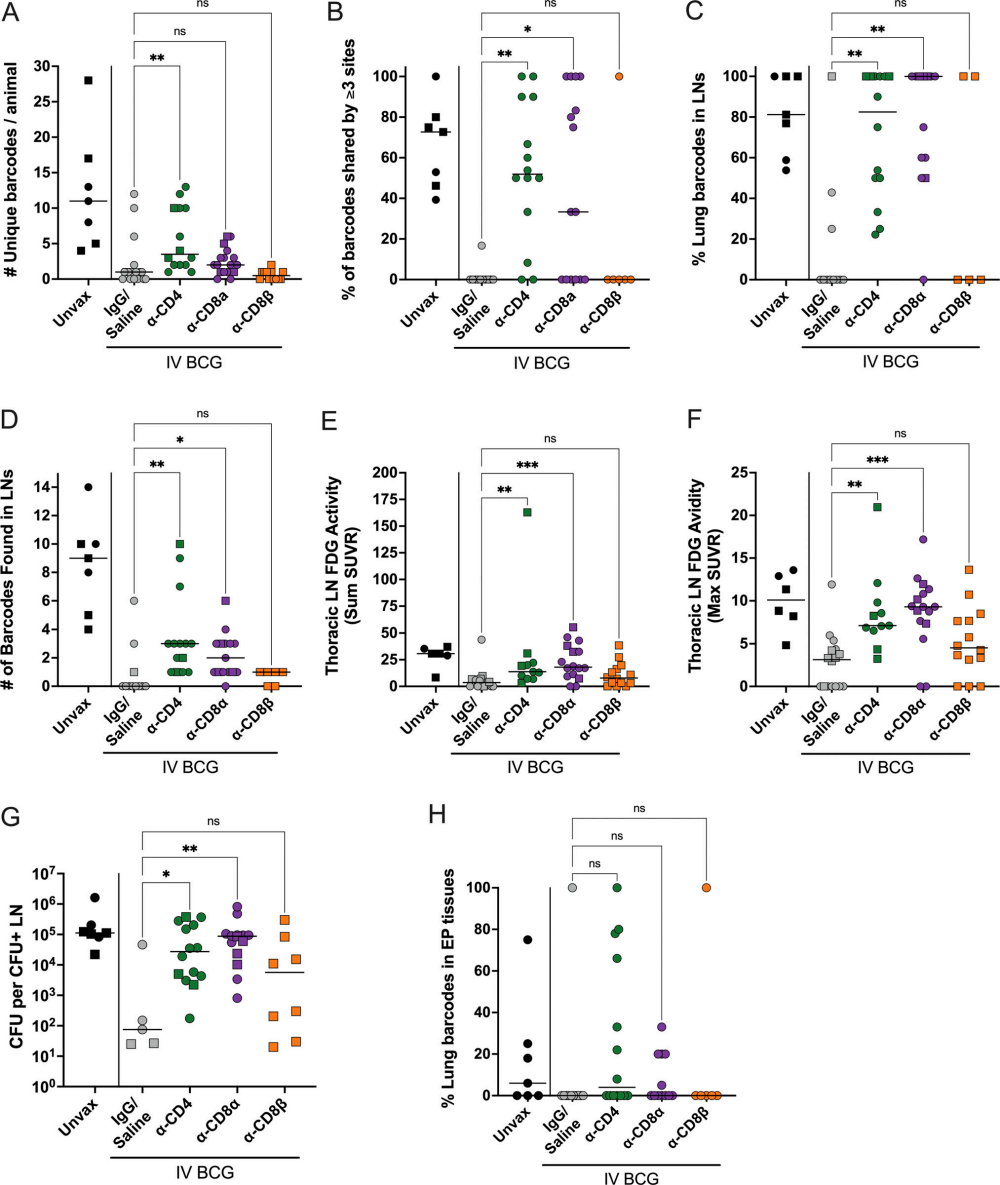
**CD4 and CD8α depletion results in increased dissemination within lung and to LNs. (A)** The total number of unique barcodes identified in each animal. Sterile animals are shown as a value of 0. Samples failing sequencing quality control were excluded from analysis. Unvax: *n* = 7; IgG/saline: *n* = 17; α-CD4: *n* = 14; α-CD8α: *n* = 17; and α-CD8β: *n* = 12. **(B)** The frequency of all established barcodes found in three or more sites, regardless of tissue. Unvax: *n* = 7; IgG/saline: *n* = 11; α-CD4: *n* = 14; α-CD8α: *n* = 15; and α-CD8β: *n* = 6. **(C)** The frequency of all barcodes found in lung tissue or granulomas that were also identified in thoracic LNs. Unvax: *n* = 7; IgG/saline: *n* = 11; α-CD4: *n* = 14; α-CD8α: *n* = 13; and α-CD8β: *n* = 5. **(D)** The number of barcodes found in any thoracic LN. Unvax: *n* = 7; IgG/saline: *n* = 11; α-CD4: *n* = 14; α-CD8α: *n* = 15; and α-CD8β: *n* = 6. Sterile animals are not included in B–D. **(E)** The total FDG activity in thoracic LNs as a sum of all measured LNs. **(F)** The single thoracic LN with maximum FDG avidity per animal. In E and F, if no LNs were distinguishable on the scan, a 0 is reported; animals missing PET scans were not included. Unvax: *n* = 6; IgG/saline: *n* = 16; α-CD4: *n* = 11; α-CD8α: *n* = 16; and α-CD8β: *n* = 14. **(G)** CFUs per non-sterile thoracic LN showing the mean of all CFU^+^ LNs in an animal. Animals with no CFU detected in LN tissues were not included. Unvax: *n* = 7; IgG/saline: *n* = 5; α-CD4: *n* = 14; α-CD8α: *n* = 14; and α-CD8β: *n* = 8. **(H)** The frequency of barcodes found in lung tissues that were also found in extra pulmonary tissues. Unvax: *n* = 7; IgG/saline: *n* = 11; α-CD4: *n* = 14; α-CD8α: *n* = 12; and α-CD8β: *n* = 6. All groups (excluding unvaccinated animals) were compared using the Kruskal–Wallis test with Dunn’s multiple comparison adjusted P value ranges indicated, comparing between IgG/saline group and each depletion group. Symbols represent an animal, line represents median of group. Circles represent cohort 1, and squares represent cohort 2. ns = not significant, P ≥ 0.05; * = 0.01 ≤ P < 0.05; ** = 0.001 ≤ P < 0.01; and *** = 0.0001 ≤ P < 0.001. SUVR: standardized uptake value ratio.

To gain further insight about Mtb infection establishment and dissemination following depletion in vaccinated macaques, we analyzed data from the genetically barcoded strain of Mtb Erdman used for infection in conjunction with serial PET CT scans to quantify how many bacilli initiated infection and the extent and sites of dissemination (Data S1) (Martin et al., 2017; Winchell et al., 2023). We previously demonstrated that each granuloma is seeded by a single bacterium (Lin et al., 2014; Martin et al., 2017). The number of unique barcodes found in vaccinated animals was reduced compared with unvaccinated animals (Fig. 8 A). Paired with minimal granuloma numbers and Mtb CFU in the vaccinated control group, these data corroborate our previous results that i.v. BCG has a robust effect on limiting Mtb establishment (Darrah et al., 2020, 2023). CD4 depletion led to a modest yet significant increase in unique barcodes compared with undepleted, vaccinated macaques, indicating a slight loss of control over establishment despite high thoracic burdens. Depletion of CD8^+^ cells by either anti-CD8α^−^ or CD8β^−^ antibodies did not result in increased numbers of unique barcodes, which aligns with the limited early (4 wk) granuloma formation observed by PET CT (Fig. 4 B).

To quantify dissemination, we evaluated the percentage of barcodes that were found in multiple sites (i.e., barcodes shared by granulomas, lung tissue, and/or LNs). Specifically, we used a cutoff of three or more shared sites to capture widespread dissemination, avoiding one-off dissemination events from granuloma to a LN that are a general feature of the macaque model. Undepleted, vaccinated macaques had no or minimal dissemination, yet both CD4 and CD8α depletion resulted in significantly increased dissemination (Fig. 8 B). Barcodes shared between a lung or lung granuloma and a LN increased in CD4- and CD8α-depleted animals, with most or all established barcodes spreading to LNs (Fig. 8 C). Unique barcodes in thoracic LNs also increased in CD4- and CD8α-depleted animals relative to undepleted, vaccinated controls (Fig. 8 D). CD8β depletion resulted in minimal dissemination, similar to undepleted, vaccinated animals.

We developed methods to quantify inflammation (via FDG avidity and PET CT scans) in the thoracic LNs (see Materials and methods). Our previously published data showed that thoracic LNs with high FDG avidity were very likely to contain Mtb bacilli (Ganchua et al., 2018). Both CD4- and CD8α-depleted macaques had significantly elevated FDG activity in LNs compared with undepleted, vaccinated controls, while the CD8β-depleted group was not significantly different than vaccinated controls (Fig. 8 E). The maximum FDG avidity, quantifying the most inflamed LN per animal, significantly increased following CD4 and CD8α depletion (Fig. 8 F). Historically, we have seen that substantially diseased and necrotic LNs can infiltrate the airway and rupture, rapidly spreading large numbers of bacilli throughout the lung. Prevention of LN disease progression by vaccines is important to limit overall disease progression.

CD4- and CD8α-depleted animals were impaired in control of Mtb dissemination to and replication in LNs (Fig. 8 G and Fig. 5 D). Mtb bacterial burden in involved LNs following either CD4 (median: 2.7 × 10^4^ CFUs) or CD8α (median: 8.7 × 10^4^ CFUs) depletion was significantly higher than undepleted, vaccinated controls (median: 75 CFUs). We note a threefold increase in median CFUs in the CD8α-depleted group compared with the CD4-depleted group, suggesting more bacterial growth in LNs in the absence of CD8α^+^ lymphocytes. However, no significant increase in barcodes shared between lung (uninvolved or granuloma) and EP tissues was found in any depletion group (Fig. 8 H).

## Discussion

i.v. BCG vaccination is highly effective against Mtb infection and disease in NHPs, providing a model to assess both immune correlates and mechanisms of protection. i.v. BCG results in a large influx of antigen-specific T cells to the airway, and our correlates analysis found that CD4 T cells producing Th1 or Th17 cytokines and the number of NK cells in the airways were primary correlates of protection in this model (Darrah et al., 2020, 2023). These data corroborate rodent and macaque models and human observational studies that demonstrate a key role for CD4^+^ T cells in anti-mycobacterial protection (Leveton et al., 1989; Lin et al., 2012; Morgan et al., 2021; Nunes-Alves et al., 2014). Of note, the expansion of mycobacteria-specific CD8^+^ T cells following i.v. BCG suggested that a multicellular mechanism could also be necessary for protection. Our study investigated the influence of depletion of CD4^+^, CD8α^+^, or CD8αβ^+^ lymphocytes on the establishment, progression, and dissemination of Mtb following i.v. BCG vaccination to identify mechanisms of protection. Our results demonstrate a requirement for CD4 T cells and CD8α^+^ lymphocytes, but not CD8αβ T cells, for i.v. BCG–induced protection in macaques.

CD4 or CD8α, but not CD8β, antibody-mediated depletion abrogated i.v. BCG–induced immunity in most animals. This supports nonredundant roles for CD4^+^ T cells and CD8α^+^ lymphocytes in protection mediated by i.v. BCG by the primary outcome measure of thoracic Mtb bacterial burden. These results are consistent with other data supporting a key protective role for T cells against Mtb infection (Bromley et al., 2024; Chen et al., 2009; Lin et al., 2012; Winchell et al., 2023). While CD4 T cells are canonically associated with both natural immunity and vaccine responses to Mtb infection (Morgan et al., 2021; Nunes-Alves et al., 2014), we recently showed that CD8^+^ lymphocytes are also critically important for controlling early Mtb infection in unvaccinated animals (Winchell et al., 2023). In i.v. BCG–vaccinated animals, we expected that, given the influx of antigen-specific CD8^+^ T cells in airways and the return of rapidly expanded γδ T cell and MAIT populations to pre-vaccination baselines, we would see similar effects on protection between the two CD8 depletion groups. Anti-CD8α antibody targets both CD8αα- and CD8αβ-expressing cells, including NK cells, γδ T cells, MAITs, and CD4^+^CD8α^+^ DP T cells, in addition to conventional adaptive CD8αβ T cells. Anti-CD8β antibody, however, primarily targets conventional CD8 T cells, which express CD8αβ heterodimers. We found that CD8α, but not CD8β, depletion diminished i.v. BCG–induced protection. Our results raise the possibility that CD8α^+^ lymphocyte subsets, CD4^+^CD8α^+^ DP T cells, or innate-like cells, like NK/ILCs, γδ T cells, or MAITs, are playing a role in BCG i.v.–induced protection either alone or in combination. This conclusion aligns with the primary correlates of protection following i.v. BCG being the number of CD4 T cells, either SP or CD4^+^CD8α^+^, and NK cells in the airways (Darrah et al., 2023; Roy Chowdhury et al., 2018). Congruence of correlates and mechanisms of protection is not guaranteed, but these data suggest the ability to directly monitor protective responses following vaccination rather than predictive measures, at least for i.v. BCG. Although antibody responses were elevated in response to i.v. BCG, a separate study in which we depleted macaques of B cells during the early vaccination phase and greatly diminished antibody levels throughout the study, demonstrated little to no effect on i.v. BCG–induced protection (Wang et al., 2024).

We have considered potential experimental factors that could confound our ability to see a loss of protection in CD8β-depleted animals. Insufficient depletion is unlikely, according to our own analysis and a recent study that clearly demonstrated the effectiveness of CD8β depletion in i.v. BCG–vaccinated but unchallenged macaques (Sutton et al., 2024). Further, CD8β depletion via antibody infusion is capable of disrupting the control of infections in other disease models (Liu et al., 2022; Mcmahan et al., 2021; Nishimura et al., 2017; Okoye et al., 2021). Therefore, our data support that the mycobacteria-specific airway or lung adaptive CD8 T cells are not required for i.v. BCG–induced early protection. It is possible that since MHC Class I primarily presents ESAT-6 secretion system 1 (ESX-1) antigens on Mtb-infected human macrophages in vitro (Leddy et al., 2023), CD8 T cells may need to recognize infected macrophages presenting ESX-1 antigens to be protective in primates. ESX-1 antigens are not expressed by BCG due to the region of deletion 1, so CD8αβ T cells recruited to the lung following i.v. BCG vaccination likely recognize mycobacterial antigens expressed by BCG that are presented less efficiently by Mtb-infected macrophages.

One interpretation of our data is that CD4 T cells and innate-like CD8α^+^ lymphocytes are both critical for i.v. BCG–induced protection. However, it could be that the removal of a singular cell type from the system is responsible for the effect seen in both groups. The most likely alternative interpretation is that depletion of CD4^+^CD8α^+^ T cells after either anti-CD4 or anti-CD8α treatment could have abrogated protection against TB, implicating these DP cells as a cellular source of i.v. BCG–mediated immunity. CD4^+^CD8α^+^ T cells have been described as both an activated CD4 subset and a distinct population (Overgaard et al., 2014; Zuckermann, 1999). A study of chronic cytomegalovirus infection in macaques demonstrated that the antigen-specific T cell population was highly enriched within the CD4^+^CD8α^+^ DP T cells relative to CD4^+^ SP T cells (Macchia et al., 2006). In that context, CD4^+^CD8α^+^ DP cells were more differentiated and functional compared with CD4 SP cells. These data implicate CD4^+^CD8α^+^ T cells in protection given the vaccine-induced expansion of T cells. However, our data do not support this effect occurring to the same extent in i.v. BCG–vaccinated macaques. The CD4^+^CD8α^+^ T cells in i.v. BCG–vaccinated animals have a more highly activated and functional response without ex vivo stimulation, while CD4^+^ SP T cells demonstrate higher antigen-specific cytokine production upon WCL stimulation. Our results are corroborated by previously published analysis of mycobacteria-specific cytokine production by CD4 SP and CD4^+^CD8α^+^ DP T cells in i.v. BCG–vaccinated, but Mtb naïve, macaques (Sutton et al., 2024). Thus, while CD4^+^CD8α^+^ T cells may be involved in i.v. BCG–induced protection, they do not comprise the majority of mycobacteria-specific T cells in the lung and are not likely to be the sole source of protection. Despite these data, the possibility of a key role for these cells cannot be ruled out without targeting them in isolation, which is outside the scope of this study and not feasible with current reagents.

A single source of protection could also be γδ T cells, the majority of which are CD8αα^+^ in granulomas. The other γδ T cells are primarily CD4^−^CD8α^−^, although some CD4^+^ γδ T cells were observed. γδ T cells previously were shown to have protective potential in macaques (Qaqish et al., 2017; Shen et al., 2002). Depletion with anti-CD8α antibody or anti-CD4 antibody did not change the total γδ T cell population, with CD4^−^CD8α^−^ γδ T cells expanding to fill the cellular and functional gap created by CD8α depletion. Thus, we conclude that while γδ T cells certainly may have a role in i.v. BCG–induced protection, the primary role may be during the initiation of the immune response, hence the short-lived expansion following vaccination, rather than during the effector phase of protection.

Our data support that one or more subsets of CD8α^+^-expressing lymphocytes, in addition to CD4 T cells, are needed for i.v. BCG–induced protection. Recent studies have shown that CD8α^+^ lymphocytes play a key role in the control of early Mtb infection in macaques (Chen et al., 2009; Winchell et al., 2023). Here, the question remains which CD8α^+^ lymphocytes are necessary for the protection. Memory-like NK cells have been shown to exhibit therapeutic potential in vaccine models (Geary and Sun, 2017; Peng and Tian, 2017). Relevant to our study, NK cells are affected by CD8α depletion, although CD8α-negative CD16^+^ and NKG2A^+^ NKs are still present after CD8α depletion. Specifically, CD8α^+^CD16^−^NKG2A^−^ were the primary population affected by depletion. The CD8α^+^ NK subsets are sources of IL-17 and TNF in the airway after i.v. BCG vaccination, cytokines that have been implicated in the control of Mtb infection (Lin et al., 2007; Torrado and Cooper, 2010). IL-17 produced specifically by innate lymphocytes has been implicated in pro-inflammatory responses in the lung and mucosal immunity against pathogenic bacteria (Gelmez et al., 2021; Lunding et al., 2015). CD8α^−^CD16^−^NKG2A^+^ cells, which produce less IL-17 and TNF, filled the void left by the loss of CD8α^+^ NK cells after depletion. NK cells may also be responsible for priming adaptive T cell responses during i.v. BCG vaccination. Another possibility to consider is that unconventional T cell subsets are playing a role in BCG i.v.-induced protection, including MAIT (MR-1 restricted) (Chengalroyen, 2023), NKT (CD1d restricted) (Arora et al., 2013), and GEM (CD1b restricted) (Siddiqui et al., 2015) T cells, all of which can express CD8α. It is important to note that there are key differences between species, such as most MAITs being CD4^−^CD8^−^ DN in mice and CD8α^+^ or CD8αβ^+^ in humans and NHPs. An early expansion of MAITs was observed following i.v. BCG, suggesting that their role may be in priming adaptive responses rather than combating the infection directly. It should also be noted that we did not have the ability to further characterize CD8αα T cell populations present in granulomas as MAITs, iNKTs, or CD1b restricted due to low event rates and therefore cannot determine whether these niche populations could contribute to protective capacity following CD8β depletion. At this time, depletion of specific innate-like or unconventional T cell subsets in macaques is not feasible due to lack of appropriate reagents. However, future studies could address this area by combining broad depletion as employed here in combination with methods such as adoptive transfer of individual populations.

Both CD4 and CD8α groups developed a small number of lesions, while still experiencing high bacterial burden. Thus, the infection establishment bottleneck in the airways was not greatly affected by CD4 or CD8α depletion, although the few Mtb that entered the lung parenchyma were uncontrolled in the absence of CD4 T cells or CD8α^+^ lymphocytes and disseminated within the lung and to the LNs. We posit that 5 mo of i.v. BCG–induced T cells in airways interacting with and activating macrophages in the presence of high levels of antigen prior to depletion is critical in priming a robust first line of defense. Single-cell RNA sequencing (scRNAseq) analysis and murine studies support a model of macrophage training that is T cell dependent and contributes to limiting establishment of infection (Kaufmann et al., 2018; Peters et al., 2023, *Preprint*). Cross-protection against infection with influenza A in mice has demonstrated the role of T cells in the generation of trained immunity following i.v. BCG (Tran et al., 2024). It is possible that there is redundancy in the activation of macrophages by these lymphocyte subsets, such that removal of one subset did not affect the bottleneck for infection.

Limitations to this study include our inability to determine which types of innate-like CD8^+^ lymphocytes contribute to i.v. BCG protection due to the lack of reagents for in vivo selective depletion of specific cell types, such as NK, NKT, γδ T cells, MAITs, and other innate subsets, in macaques. In the current study, we did not include depletion of the CD4 and CD8 subsets in unvaccinated animals, but we and others have reported on this previously (Bromley et al., 2024; Chen et al., 2009; Lin et al., 2012; Winchell et al., 2023). In addition, the small number of granulomas obtained from the CD8β-depleted group limited our analysis of these samples. Antibody depletion is not complete, as knock-out systems are, but we have shown >90% depletion of target populations in multiple tissue sites. Finally, the level of protection due to high-dose i.v. BCG in our previous experiments was extremely high, with 90% protection (<100 Mtb CFUs/animal) (Darrah et al., 2020, 2023). During analysis of lymphocyte function, we used WCL as a broad mycobacterial (including BCG) stimulator and ESAT6^+^CFP10 peptides to elicit Mtb (excluding BCG)-specific responses. WCL is more effective in eliciting CD4 responses than CD8 responses. We have previously tested peptide pools thought to more effectively elicit CD8 responses; however, none have outperformed WCL in our samples thus far. As such, antigen-specific cytokine profiles of certain cell types may be limited. In this study, we used the same strain and cryopreserved culture stock of BCG (Staten Serum Institute), and although there was still a 10,000-fold reduction in Mtb burden compared with unvaccinated animals in the undepleted BCG i.v. group; we did not obtain the same overall level of sterilizing immunity that we saw previously. The reasons for this are unclear, as there were no experimental differences noted in the vaccination procedure and, while slightly below that described previously, the true BCG dose was well above that required for protection (Darrah et al., 2020, 2023). Further, the Mtb challenge dose (∼17 CFUs) was slightly higher than the previous studies (∼10 CFUs), although this is still within the standard range of inoculum doses used. The cause might be, and likely is, as simple as statistical variation, as the first study had only 10 NHPs per group, and rhesus macaques are an outbred animal model and are obtained from various sources. Lastly, in our interpretation of the data, this model is not necessarily indicative of protective mechanisms in natural infection (i.e., reinfection or reactivation). Further studies are required to assess such questions, which should offer an interesting balance in informing targets of future vaccine candidates.

In conclusion, these results support multiple, nonredundant cell-mediated mechanisms of protection involved in protection against TB provided by i.v. BCG. Further work must still be done to elucidate the critical CD8αα^+^ subset(s) that are complementary to CD4 T cells in conferring robust protection by i.v. BCG vaccination. The findings here may be relevant for defining successful vaccine response metrics in future candidate screening. Canonical CD4 T cell responses are important in combating Mtb, as we and others have found, but they are independently incapable of effective control in the absence of their innate-like CD8^+^ counterparts, at least with i.v. BCG. This model system provides significant insight into key cellular mechanisms that can be used to evaluate future candidates.

## Materials and methods

### Animals and handling

This research involved the use of Indian-origin rhesus macaques (*M. mulatta*), between 3 and 7 years of age. Cohort 1 (*n* = 40) was split evenly between males and females (*n* = 20 each), while cohort 2 (*n* = 32) was all males due to limited availability. All experimental procedures involving care of animals complied with ethical regulations at the respective institutions (Animal Care and Use Committees of the Vaccine Research Center, National Institute of Allergy and Infectious Diseases, National Institutes of Health (NIH), and of Bioqual, Inc., and of the University of Pittsburgh School of Medicine Institutional Animal Care and Use Committee). Macaques were housed and cared for in accordance with local, state, federal, and institute policies in facilities accredited by the American Association for Accreditation of Laboratory Animal Care, under standards established in the Animal Welfare Act and the Guide for the Care and Use of Laboratory Animals as mandated by the U.S. Public Health Service Policy. Macaques were monitored for physical health, food consumption, body weight, temperature, complete blood counts, and serum chemistries. Vaccinations and pre-challenge blood draws and BALs were performed at the NIH and Bioqual. Animals were transported to the University of Pittsburgh for Mtb challenge and analyses. All Mtb infections were performed a Biosafety Level 3 (BSL3) facility. Veterinary staff regularly monitored clinical signs following challenge, including appetite, behavior and activity, weight, erythrocyte sedimentation rate, Mtb growth from gastric aspirate, and coughing. Input from these examinations, along with serial PET-CT imaging, were used to determine whether a macaque met criteria for the humane end point before the predetermined study end point of 8 wk after infection.

### Sample size and statistics

The sample size for this study was determined using bacterial burden (measured as log_10_-transformed total thoracic CFUs) as the primary outcome variable. Comparisons included in the analysis were between the undepleted, vaccinated controls (IgG/saline) and each depletion group (anti-CD4, anti-CD8α, and anti-CD8β). A standard deviation of 1.8 log_10_ CFU was conservatively estimated from prior studies using BCG vaccination (Darrah et al., 2020, 2023). Using this standard deviation, the group sizes provide power to detect a mean difference of 2.5 log_10_ CFU for two-sided *t* tests between undepleted immunized controls and CD4-depleted animals (87.3%), CD8α-depleted animals (92.9%), and CD8β-depleted animals (87.3%) with an alpha = 0.05/3 = 0.0167 to account for three pairwise comparisons. Unvaccinated infection controls were included in each challenge cohort to ensure Mtb infection was achieved, but these animals were not included in either the power analysis when planning the study or the statistical tests run on the data. In our previous data, log_10_-transformed total thoracic CFUs follow a normal distribution. Skewed distributions caused by protected animals made it necessary to analyze the data from the study using unplanned nonparametric methods.

For analyses, depletion groups were compared with vaccinated IgG/saline controls using the Kruskal–Wallis test, with Dunn’s multiple comparison-adjusted P values shown. When comparing samples from the same animal within group (across time points or cell types), Wilcoxon matched-pairs signed-rank test was used. To compare cell type responses after vaccination, Tukey’s test adjusting for multiple comparison was used to compare each time point to baseline levels. Presence of stimulation-specific T cells was tested using one-sample *t* tests (testing if mean is equal to 0). All statistical tests were run in GraphPad Prism for macOS (version 10.1.1). For numbers of granulomas found at necropsy and counted on PET CT, areas of TB pneumonia and consolidations were considered to be too numerous to count and numerically represented as 100. For all graphs with zero values that were log_10_-transformed, the number 1 was added to the entire data set.

### Vaccination

Macaques were randomized into vaccinated (*n* = 65) and unvaccinated (*n* = 7) groups based on age, weight, and gender. The macaques were vaccinated under sedation. Cryopreserved aliquots of BCG Danish Strain 1331 (Statens Serum Institute, Copenhagen, Denmark) were thawed immediately before vaccination and diluted in cold PBS containing 0.05% tyloxapol (Sigma-Aldrich) to a target dose of 5 × 10^7^ CFUs. The solution was delivered i.v. into the saphenous vein in a volume of 2 ml. Actual BCG doses were quantified by dilution plating and are reported in Table S1.

### Lymphocyte depletion

Vaccinated macaques were randomly assigned to four depletion groups. IgG (*n* = 6) and saline (*n* = 12) served as a combined undepleted control group, with anti-CD4 (*n* = 16), anti-CD8α (*n* = 17), and anti-CD8β (*n* = 14) as experimental groups. The Anti-CD4 [CD4R1] antibody (NIH Nonhuman Primate Reagent Resource Cat#PR-0407, RRID:AB_2716322), Anti-CD8 alpha [MT807R1] antibody (NIH Nonhuman Primate Reagent Resource Cat#PR-0817, RRID:AB_2716320), and Anti-CD8 beta [CD8b255R1] antibody (NIH Nonhuman Primate Reagent Resource Cat# PR-2557, RRID:AB_2716321) were engineered and produced by the Nonhuman Primate Reagent Resource. Starting 5 mo after vaccination and 4 wk prior to Mtb challenge, the macaques were administered depletion antibodies at 50 mg/kg/dose i.v. every 2 wk, continuing through Mtb challenge until necropsy, as previously described (Bromley et al., 2024; Sutton et al., 2024; Winchell et al., 2023). Cohort 1 did not include any macaques assigned to CD8β depletion. Cohort 2 consisted of primarily CD8β-depleted animals, with an additional 4 animals in each of the other groups as internal controls for any cohort effect (Table S1).

### Infection

Examination of animals was performed in quarantine upon arrival at the University of Pittsburgh to assess physical health and confirm no previous Mtb infection was detected via ELISpot assays.

All macaques were challenged via bronchoscopic instillation with 5–39 CFUs of barcoded Mtb (Erdman), as previously described (Table S1) (Capuano et al., 2003; Lin et al., 2009). This range of doses has resulted in comparable levels of progressive TB in unvaccinated rhesus macaques in this and previous studies (Darrah et al., 2020; Maiello et al., 2018). Infection groups included animals from multiple experimental groups to reduce dose bias.

### PBMCs, BAL, and LN biopsy processing

PBMCs were isolated from whole blood draws using Ficoll-Paque PLUS gradient separation, as previously described (Darrah et al., 2019). BAL samples were centrifuged, and the supernatant was collected and frozen. Remaining cells were resuspended in warm R10 (RPMI 1640 with 2 mM L-glutamine, 100 U ml^–1^ penicillin, 100 μg ml^–1^ streptomycin, and 10% heat-inactivated FBS). Biopsied LNs were mechanically disrupted and filtered through a 70-μm cell strainer. Single-cell suspensions were resuspended in warm R10 to be counted and for use in same-day assays. If necessary, cells were cryopreserved in FBS containing 10% DMSO between 5 × 10^6^ and 2 × 10^7^ cells ml^−1^.

### Electrochemiluminescent antibody ELISA

IgG, IgM, and IgA were batch analyzed from cryopreserved 10× concentrated BAL (in PBS) or plasma. A 384-well MA6000 high-binding plate (MSD L21XB) was washed with PBS/Tween 20 (0.05%) and coated with 5–10 µg/ml H37Rv Mtb WCL at 4C overnight. All reagents were brought to room temperature (RT), and plates were washed prior to blocking with MSD Blocking Buffer A (5% BSA) for 1 h at RT (shaking, 900 rpm) and then washed. Samples were diluted (1:20 and then serially 1:5 for a total of 7) in diluent 100 (MSD), added to plates, and incubated with shaking (900 rpm) for 2 h at RT, then washed. MSD-Sulfo-Tag-Conjugated anti-hu/NHP secondary antibodies (0.5 µg/ml; IgG cat#D20JL-6, IgA cat#D20JJ-6, and IgM cat#D20JM-6) for 1 h at RT (900 rpm) and washed. Gold Read Buffer A (HB) was added, plates were run on the MSD Meso Sector S 600 MM reader, and analyzed using Methodical Mind software v2.0.5. AUC (ECL, arbitrary units) of individual binding curves for each sample were calculated in Prism v10.1.1.

### ELISpot assays

IFN-γ ELISpot assays were performed, as previously described, after vaccination prior to depletion, following initiation of depletion infusions before Mtb challenge, and at necropsy (Darrah et al., 2020). Hydrophobic high protein-binding membranes 96-well plates (Millipore Sigma) were hydrated with 40% ethanol, washed with sterile water, and coated with anti-human/monkey IFN-γ antibody (15 μg ml^−1^, MT126L; MabTech) overnight at 4°C. Plates were washed with PBS and blocked with R10 media for 2 h at 37°C with 5% CO_2_. 2 × 10^5^ PBMCs per well were incubated in R10 media only or containing 1 μg ml^−1^ each of ESAT-6 or CFP-10 peptide pools (BEI Resources) for 40–48 h. To develop, plates were washed with PBS and biotinylated anti-human IFN-γ antibody (2.5 μg ml^−1^, 7-B6-1; MabTech) was added for 2 h at 37°C. After washing, streptavidin-HRP (1:100; MabTech) was added for 45 min at 37°C. Spots were stained using AEC peroxidase (Vector Laboratories, Inc.) and counted on a plate reader. Assays were performed using frozen PBMCs thawed 1 day prior to the assay start and rested in R10 media at 37°C overnight. Assays were validated using phorbol 12,13-dibutyrate (PDBU) and ionomycin (I; together: P+I) as a positive control stimulation condition, guaranteeing cellular functionality after a freeze/thaw cycle. If no spot formation was observed with P+I stimulation, the assay was repeated with a different frozen vial. Results at given time points are not reported from animals for which the assay was never successful.

### Necropsy procedures and tissue processing

Macaques were euthanized by sodium pentobarbital injection and necropsied between 7 and 11 wk after challenge, as previously described (Darrah et al., 2020; Gideon et al., 2022; Lin et al., 2014; Martin et al., 2017). Gross pathology observed in lungs (including number and size of granulomas, consolidations, and pneumonias), LNs (including swelling, lesions, and necrosis), and EP compartments (such as peripheral LNs, spleen, liver, and gastrointestinal tract) was quantified using a published scoring system (Lin et al., 2009; Maiello et al., 2018). Individual lesions were identified by PET-CT mapping before excision. These tissues, along with regions of uninvolved lung tissue from each of the seven lobes, were excised and homogenized into single-cell suspensions for CFU quantification, barcoding analysis, scRNAseq, and various immune assays. Homogenization was accomplished by mechanical disruption and passing the suspension through a 70-μm cell strainer. Lung lobes and other tissues designated for scRNAseq were subjected to enzymatic dissociation (GentleMACS; Miltenyi). Sections of lung lodes, thoracic LNs, and large granulomas (>2 mm in diameter) were also processed for formalin fixation and paraffin embedding for histological analysis.

### Bacterial burden quantification

CFUs were calculated from plating serial dilutions of homogenized tissues excised during necropsy on 7H11 agar plates, as previously described. Quantification of bacterial burden was performed as previously described (Lin et al., 2009). Plates were incubated for 21 days at 37°C in 5% CO_2_ before CFU counting.

### Mtb CFUs and barcode determination

To track the establishment and dissemination of Mtb, barcoded Mtb Erdman was used for infection and barcodes were determined after necropsy as previously described (Martin et al., 2017). A library of digitally barcoded plasmids containing 7-mer barcodes and adjacent 74-mer library identifier sequences was stably transformed into the bacterial chromosome of Mtb Erdman. Each library contained ∼16,000 unique barcodes, and three independently generated libraries were combined into a master library to increase barcode diversity, thereby ensuring a <2% chance that a barcode would be represented twice if 20 bacteria were randomly selected. Mtb colonies on plates from necropsy tissues were DNA extracted using phenol–chloroform methods. After DNA purification, samples were subjected to amplicon-based sequencing to identify all the barcode tags present and shared across tissues. Q-tags and barcodes were identified and quantified as previously described (Winchell et al., 2023). Distribution of barcodes in CFU^+^ tissues is shown in Data S1.

### Flow cytometry

**Figure S4.**
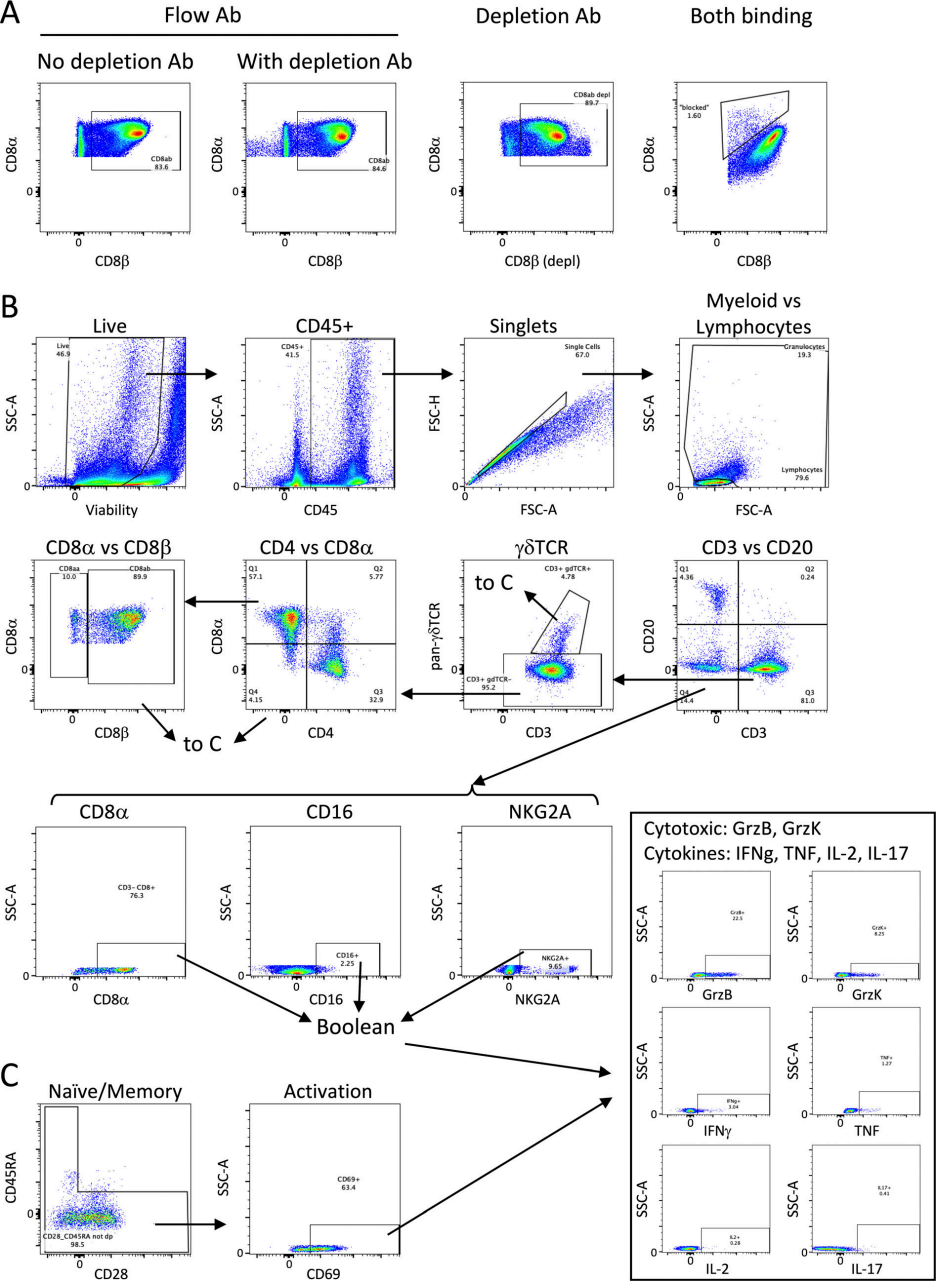
**Gating strategy for flow cytometry samples. (A)** CD8β staining by flow cytometry is not inhibited by anti-CD8β depletion antibody. PBMCs stained with or without the addition of the depletion a-CD8β antibody show similar levels of staining by the antibody used for flow cytometry, indicating negligible competitive blocking. **(B and C)** Representative phenotype (B) and memory and effector function (C) gating on lung tissue from a vaccinated, undepleted animal.

**Figure S5.**
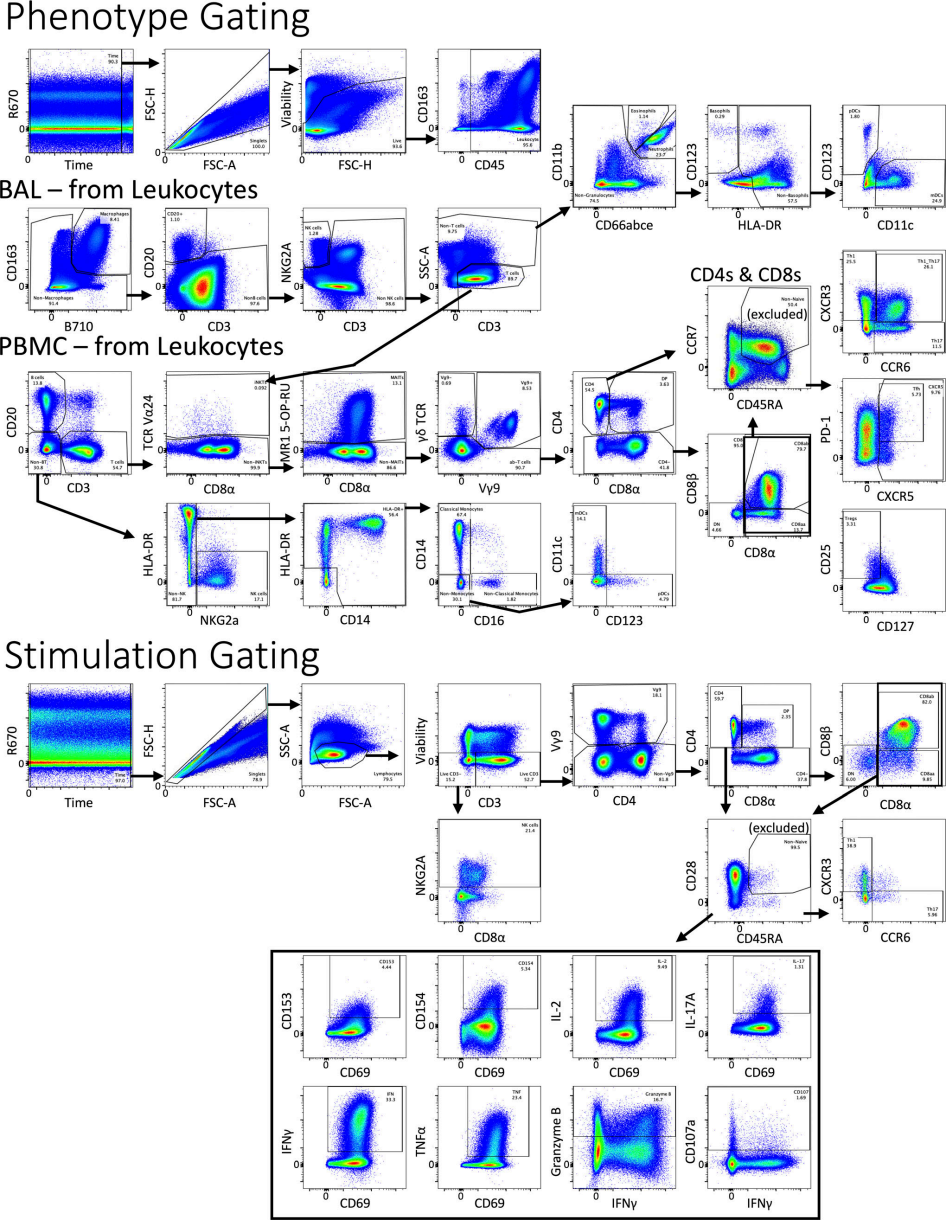
**Gating strategy for BAL and PBMC phenotyping flow cytometry samples.** Representative BAL and PBMC samples from an i.v. BCG–vaccinated animal at a peak time point.

Flow cytometry was performed on BALs prior to Mtb challenge, PBMCs throughout the study, and a subset of lung lobes, LNs (peripheral and thoracic), and granulomas processed at necropsy. Additionally, cryopreserved PBMCs were processed as a batch. Immediately after processing, up to 5 × 10^6^ cells from single-cell suspensions allocated for intracellular cytokine staining were stimulated by incubation with R10 media only, 20 μg ml^−1^ H37Rv Mtb WCL, or 1 μg ml^−1^ each of ESAT-6 and CFP-10 peptide pools for 2 h at 37°C in 5% CO_2_. Necropsy tissue samples were then incubated with 10 μg ml^−1^ BD GolgiPlug (BD Biosciences) overnight for up to 12 h. BAL and PBMC samples were incubated with either GolgiPlug or 1:500 eBioscience Protein Transport Inhibitor Cocktail (500×) (Life tech) for 6–12 h. After stimulation and blocking, samples were stained with a fixable viability dye, followed by antibody panels for surface markers and intracellular antigens using standard protocols (Darrah et al., 2020). Ten PBMC samples spanning all groups, including unvaccinated, were not stained with viability dye at the 10-wk time point. Staining panels are detailed in Table S2. We confirmed our CD8β flow cytometry antibody was not being blocked by the depletion antibody, which would skew our ability to detect and characterize remaining cells (Fig. S4). Cytometry was performed on a five laser Aurora with SpectroFlo software (16UV-16V-14B-10YG-8R; Cytek) and an LSR Fortessa X-50 Cell Analyzer with DiVA software. Analysis of flow cytometry data was performed in FlowJo (v10.9.0) with gating strategy shown in Fig. S5. Unless otherwise noted, T cell subsets are γδTCR-negative. NK cells in BAL and necropsy tissues are defined as CD20^−^CD3^−^ cells that are CD8α^+^, CD16 (FcγRIIIa)^+^, and/or NKG2A (CD159a)^+^. Samples with <100 total lymphocyte events were excluded from associated frequency analysis to avoid skewing, but still included for cell count. For analysis of specific cell types (e.g., cytokine production by CD4 T cells), samples with <50 events in parent cell type gate (e.g., CD4 T cells) were excluded for frequency calculations but included for cell counts.

### PET-CT scanning

We obtained PET-CT images with a Mediso MultiScan LFER 150 integrated preclinical PET CT (Sarnyai et al., 2019). Scans were obtained following Mtb infection at 4 and 8 wk. Prior to each scan, animals were weighed and injected with a 3–5 mCi dose of PET tracer FDG. FDG is a glucose analog that collects nonspecifically in metabolically active tissue, tracking overall inflammation (Grant et al., 2022; Lin et al., 2013). Prior to obtaining PET scans, an uptake period of 50 min was observed to allow the FDG to be taken up by active tissue. In the intervening time, animals were intubated and anesthetized. CT scans were obtained during the uptake period. During CT acquisition (about 40 s in duration), the animal’s breath was held via a mechanical ventilator. This step ensures a clear CT image of the lungs.

All imaging was performed according to biosafety and radiation safety requirements within the BSL3 facility at the University of Pittsburgh. Scans were analyzed using OsiriX DICOM viewer (Rosset et al., 2004) by in-house trained PET-CT analysts (Coleman et al., 2014; Diedrich et al., 2020; White et al., 2017).

### PET-CT analysis

Measurements described below were detailed previously (White et al., 2017). “Total lung FDG activity (standardized uptake value ratio)” is calculated as the sum of all PET signal contained in the lungs above a background standard uptake value (SUV) threshold of 2.3 and divided by the average PET signal in an area of back muscle directly adjacent to the vertebrae. The division by PET uptake in resting muscle is included to account for variations in baseline metabolic activity between animals. “Number of granulomas” gives the total number of TB lesions observed in the lungs via CT. For thoracic LNs observed via PET to have a peak SUV ≥ 5, “thoracic LN FDG activity” is the sum of all the peak PET signal values measured in each thoracic LN. As in the total lung FDG activity, the summed SUV values are divided by the average PET signal from resting back muscle. This measurement represents the total metabolic activity of the thoracic LNs for each animal.

### Online supplemental material

Fig. S1 shows the changes in the composition of lymphocyte cell types in blood and airways of macaques after IV BCG vaccination and subsequent depletion. Fig. S2 shows the depletion of lymphocyte cell types. Fig. S3 shows the lymphocyte composition in granulomas and functional profiles. Figs. S4 and S5 show the flow cytometry gating trees for various main figures. Table S1 provides information related to the NHP vaccination, depletion, and infection. Table S2 provides details of flow cytometry staining panels for relevant figures. Data S1 provides the relative abundance of barcodes detected in CFU^+^ tissues.

## Supplementary Material

Table S1 shows the NHP vaccination, depletion, infection, and outcomes.

Table S2 shows the antibodies used for spectral flow cytometry.

Data S1 shows the relative abundance of detected barcodes in CFU^+^ tissues.

## Data Availability

Data are available in the main text, supplementary materials, or through IMPAc-TB’s SEEK database housed at MIT (https://fairdomhub.org/studies/1283).
